# In Vivo and In Silico Investigation Into Mechanisms of Frequency Dependence of Repolarization Alternans in Human Ventricular Cardiomyocytes

**DOI:** 10.1161/CIRCRESAHA.115.307836

**Published:** 2016-01-21

**Authors:** Xin Zhou, Alfonso Bueno-Orovio, Michele Orini, Ben Hanson, Martin Hayward, Peter Taggart, Pier D. Lambiase, Kevin Burrage, Blanca Rodriguez

**Affiliations:** From the Department of Computer Science, BHF Centre of Research Excellence, University of Oxford, Oxford, United Kingdom (X.Z., A.B.-O., K.B., B.R.); Institute of Cardiovascular Science, University College London, London, United Kingdom (M.O., P.T., P.D.L.); Mechanical Engineering Department, University College London, London, United Kingdom (B.H.); The Heart Hospital, University College London Hospital, London, United Kingdom (M.O., M.H., P.T., P.D.L.); and ACEMS ARC Centre of Excellence and School of Mathematical Sciences, Queensland University of Technology, Brisbane, Queensland, Australia (K.B.).

**Keywords:** calcium, calibration, electrophysiology, pericardium, sarcoplasmic reticulum

## Abstract

**Rationale:**

Repolarization alternans (RA) are associated with arrhythmogenesis. Animal studies have revealed potential mechanisms, but human-focused studies are needed. RA generation and frequency dependence may be determined by cell-to-cell variability in protein expression, which is regulated by genetic and external factors.

**Objective:**

To characterize in vivo RA in human and to investigate in silico using human models, the ionic mechanisms underlying the frequency-dependent differences in RA behavior identified in vivo.

**Methods and Results:**

In vivo electrograms were acquired at 240 sites covering the epicardium of 41 patients at 6 cycle lengths (600–350 ms). In silico investigations were conducted using a population of biophysically detailed human models incorporating variability in protein expression and calibrated using in vivo recordings. Both in silico and in vivo, 2 types of RA were identified, with Fork- and Eye-type restitution curves, based on RA persistence or disappearance, respectively, at fast pacing rates. In silico simulations show that RA are strongly correlated with fluctuations in sarcoplasmic reticulum calcium, because of strong release and weak reuptake. Large L-type calcium current conductance is responsible for RA disappearance at fast frequencies in Eye-type (30% larger in Eye-type versus Fork-type; *P*<0.01), because of sarcoplasmic reticulum Ca^2+^ ATPase pump potentiation caused by frequency-induced increase in intracellular calcium. Large Na^+^/Ca^2+^ exchanger current is the main driver in translating Ca^2+^ fluctuations into RA.

**Conclusions:**

In human in vivo and in silico, 2 types of RA are identified, with RA persistence/disappearance as frequency increases. In silico, L-type calcium current and Na^+^/Ca^2+^ exchanger current determine RA human cell-to-cell differences through intracellular and sarcoplasmic reticulum calcium regulation.

## Abstract 2

Supplemental Digital Content is available in the text.

Repolarization alternans (RA) are stable beat-to-beat oscillations between subsequent action potentials (APs) and are considered as an important risk factor for arrhythmogenesis.^1–3^ The mechanisms underlying RA have been the focus of extensive investigations to unravel their causes, modulators, and implications for arrhythmias such as ventricular and atrial fibrillation.^1^ However, the majority of previous studies have been conducted on animal species including rat, rabbit, cat, and dog, and therefore translation to human is compromised by interspecies differences in electrophysiology and calcium handling.

RA at fast pacing rates are known to be promoted by AP duration (APD) prolongation and steep restitution, through beat-to-beat fluctuations in ionic current availability.^4–6^ However, APD alternans have also been observed clinically in the absence of APD prolongation and without steep APD restitution curve.^7^ The new paradigm based on animal studies is now supporting that APD alternans may be caused by fluctuations in calcium-handling processes in the individual myocyte.^1,8–10^

**Editorial, see p 184**

Important issues on APD alternans mechanisms still remain unresolved particularly in human. First, the disturbances in the Ca^2+^ regulatory system responsible for calcium and APD alternans are likely to be multifactorial and modulated by a combination of Ca^2+^ transport processes. The most likely mechanism points toward Ca^2+^ alternans caused by fluctuations in sarcoplasmic reticulum (SR) Ca^2+^ content (rat experimental study)^11^ and refractoriness in ryanodine receptors (RyR; rabbit isolated cell and whole-heart experimental studies),^12,13^ with modulating factors also including the strength of Ca^2+^ reuptake through SR Ca^2+^ ATPase pump (SERCA; guinea pig experimental study).^14,15^ The mechanisms in human ventricular myocytes are, however, unknown.

Second, the key mechanisms translating Ca^2+^ alternans to APD alternans in human ventricular myocytes still need to be identified. A large calcium transient would have opposite effects on L-type calcium current (I_CaL_; through its calcium-dependent inactivation) and Na^+^/Ca^2+^ exchanger current (I_NaCa_; through the potentiation of its forward mode). Therefore, the relative effect of intracellular calcium on both currents would determine whether a large calcium transient results in long or short APD. The balance between I_CaL_ and I_NaCa_ during repolarization may differ in human ventricular cardiomyocytes with respect to other species, and cell-to-cell differences in conductances and permeabilities may modulate their role in APD alternans.

A key challenge in resolving these issues is the interpretation of findings from different animal species and cell types, and also obtained using different experimental conditions and interventions that, by aiming to segregate individual components, perturb the cellular system as a whole, depriving it of the integral phenomenon, as discussed by Valdivia.^16^ Furthermore, even careful studies performed with consistent cell types and experimental conditions exhibit differences both in the manifestation of cardiac alternans and their potential underlying mechanisms.^12^ Species differences and cell-to-cell variability in sarcolemmal conductances and permeabilities determine repolarization differences in a dynamic process modulated by internal and external factors (such as neuronal stimulation and circadian rhythms),^17–19^ which is likely to also determine cell-to-cell differences in the propensity in APD alternans generation.

The aim of this study is 2-fold: (1) to characterize potential frequency-dependent differences in APD alternans in vivo in human, and (2) to investigate the role of variability in ionic conductances and permeabilities in determining the different types of APD alternans identified in vivo using in silico human ventricular models. We hypothesize that in human ventricular cardiomyocytes, cell-to-cell variability in I_CaL_ and I_NaCa_ can explain the different types of APD alternans generation identified in in vivo recordings. We first characterize in vivo human APD alternans properties using electrophysiological recordings acquired for 6 stimulation frequencies at 240 sites of the epicardium of 41 human ventricles. To investigate the ionic mechanisms underlying human cell-to-cell differences in the occurrence and type of APD alternans, the in vivo recordings are used to construct an in silico population of biophysically detailed models of human ventricular APs, sharing the same equations but with differences in ionic protein densities to mimic cell-to-cell variability (as previously).^20–22^ Both our in silico and in vivo studies show the same 2 types of APD alternans occurring in human ventricular cardiomyocytes, characterized by an Eye-type and a Fork-type APD restitution curve, with alternans disappearing and remaining at increasingly fast frequencies, respectively. For all in silico human alternans models, SR Ca^2+^ alternans are the primary cause of both types of APD alternans, which are strongly correlated by the balance of sarcolemmal calcium currents at all frequencies. Strong I_CaL_ is responsible for the disappearance of the Eye-type alternans at fast frequencies, because of the potentiation of SERCA caused by frequency-dependent Ca^2+^ overload. I_NaCa_ is the main driver of the calcium to membrane voltage translation of alternans in the human models, and therefore blocking I_NaCa_ regulates sarcolemmal calcium balance (SCB) and suppresses alternans generation.

## Methods

### In Vivo Data Acquisition

The patient cohort consisted of 41 patients, 32 men and 9 women, aged (mean±SD) 63±13.8 years. Thirty-one patients were having coronary artery bypass grafts (24 men and 7 women); 6 patients were having aortic valve replacement (4 men and 2 women); 4 patients were having coronary artery grafts+aortic valve replacement (4 men). The subjects were selected at random from the waiting list without specific selection criteria. The study, according to the principles expressed in the Declaration of Helsinki, was approved by the local Hospital Ethics Committee, and written informed consent was obtained from all patients before the study. During cardiac surgery, a multielectrode sock was fitted over the epicardium of both ventricles, and unipolar electrograms were recorded from 240 electrodes. Ventricular pacing was established over a range of 6 cycle lengths (CLs), from 600 to 350 ms, in steps of 50 ms.^23,24^

### Activation Recovery Interval Signal Analysis

**Figure 1.**
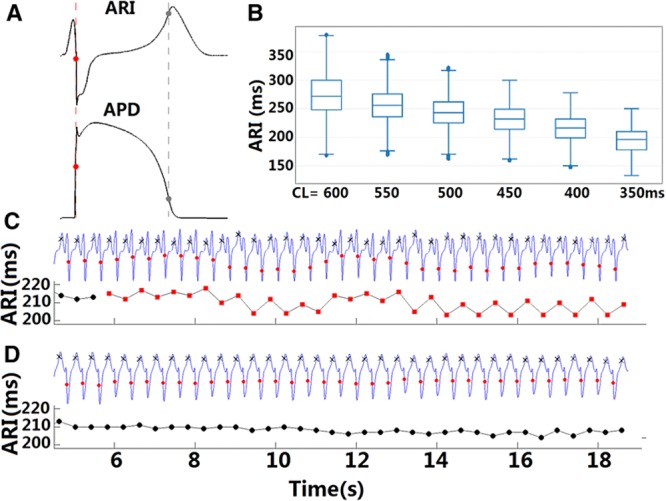
**In vivo recordings of activation recovery interval (ARI) used for the calibration of the population of human ventricular models**. **A**, ARI as an in vivo surrogate of action potential duration (APD). Red dots represent activation and depolarization times, whereas gray dots represent recovery and repolarization times. Adapted from Potse et al^25^ with permission of the publisher. Copyright ©2009, the American Physiological Society. **B**, Rate dependence and variability in in vivo ARIs aggregated from the different patients as a function of decreasing pacing cycle length (CL). **C**, Unipolar electrograms and corresponding sequence of ARIs from an alternans-susceptible site. **D**, Unipolar electrograms and corresponding sequence of ARIs from an alternans-resistant site. Dots and crosses on the electrograms represent activation and recovery times, respectively.

Activation recovery intervals, an in vivo surrogate of APD,^26^ were calculated from the epicardial electrograms as the interval from the minimum derivative during depolarization and the maximum derivative during repolarization (Figure 1A), using custom-written routines in MATLAB (MathWorks, Natick, MA). In vivo activation recovery intervals at the different CLs presented rate dependence and variability (Figure 1B), and both normal and alternans sites were observed in these patients (Figure 1C and 1D). Additional details of the in vivo activation recovery interval analysis are included in the Online Data Supplement.

### In Silico Population of Human Ventricular Models

In vivo investigations of the ionic mechanisms of cardiac alternans direct in human hearts are currently not possible. An in silico study was, therefore, performed, which, first, captured the variability in APD rate dependence from the in vivo recordings, and, second, allowed identification of key likely human ionic properties and mechanisms in RA generation. The biophysically detailed O’Hara–Rudy (ORd) model of human ventricular cell electrophysiology was adopted as the basis for the in silico investigations.^27^ The ORd model is currently considered the gold standard for human studies of proarrhythmia as it is the only one including a description of the main human ionic currents and Ca^2+^ subsystem constructed and extensively validated based on recordings >140 human hearts. Importantly, as shown in Online Table I, the ORd is the only model to include a detailed description based on human data for (1) voltage and Ca^2+^-dependent inactivation of the L-type Ca^2+^ current; (2) troponin and Ca^2+^/calmodulin-dependent protein kinase II buffering; (3) SR compartmentation; and (4) human Na^+^, Ca^2+^, and voltage dependence of Na^+^/Ca^2+^ exchanger.

To investigate the implications of variability in conductances and permeabilities in human APD rate dependence, we constructed an in silico population of human ventricular cardiomyocytes models calibrated with the in vivo recordings. First, an initial population of 10 000 human AP models was generated with models sharing the same equations, but with cell-to-cell differences in the most important conductances and permeabilities, using Latin Hypercube Sampling.^28^ Variability was considered for fast Na^+^ channel conductance, Ca^2+^ channel permeability (referred to as G_CaL_ in this study), Ks channel conductance, K1 channel conductance, Kr channel conductance, transient outward potassium channel conductance, late Na^+^ channel conductance, Na^+^/Ca^2+^ exchanger conductance, Na^+^/K^+^ pump activity, Ca^2+^ release permeability via RyR to cytoplasm, and Ca^2+^ uptake permeability via SERCA from the cytoplasm. The initial assumption to be tested by using this population is that cell-to-cell variability in protein density (rather than kinetics) is sufficient to explain differences in APD alternans generation from the in vivo recordings.

As in the study by Britton et al,^22^ a range of variation of ±100% from their original value was considered to ensure both overexpression and reduction of conductances and permeabilities. The ±100% range is necessarily an assumption as it cannot be measured in vivo, and voltage clamp data are conducted in isolated cells affected by an aggressive isolation procedure.^29^

### Calibration of the Human In Silico Models Population

The calibration of the in silico human population aimed to select the models yielding APDs with properties in range with the in vivo human recordings for 6 CLs as explained in the Online Data Supplement. Although in vivo recordings include the effects of gap junctional coupling, computer simulations using the ORd model comparing homogenous tissue and single-cell simulations have revealed both negligible differences in APD and consistency in alternans generation in single cell and tissue.^27^ Therefore, we used single-cell computer simulation studies to maximize the computational efficiency of the study and to focus on subcellular to cellular mechanisms of alternans.

### Numerical Simulations and Statistical Analysis

All numerical simulations were performed using the open source simulation software Chaste.^30^ Statistical analysis was performed using MATLAB. The Mann–Whitney *U* test was used to determine statistical differences in parameters and biomarkers. Partial correlation was used to determine the relationship between biomarkers and parameters. Pearson correlation was used to calculate the correlations in the study.

## Results

### Population of In Silico Human Ventricular Models Mimics APD Variability in the In Vivo Recordings and Identifies Key Properties Underlying Alternans Generation

**Figure 2.**
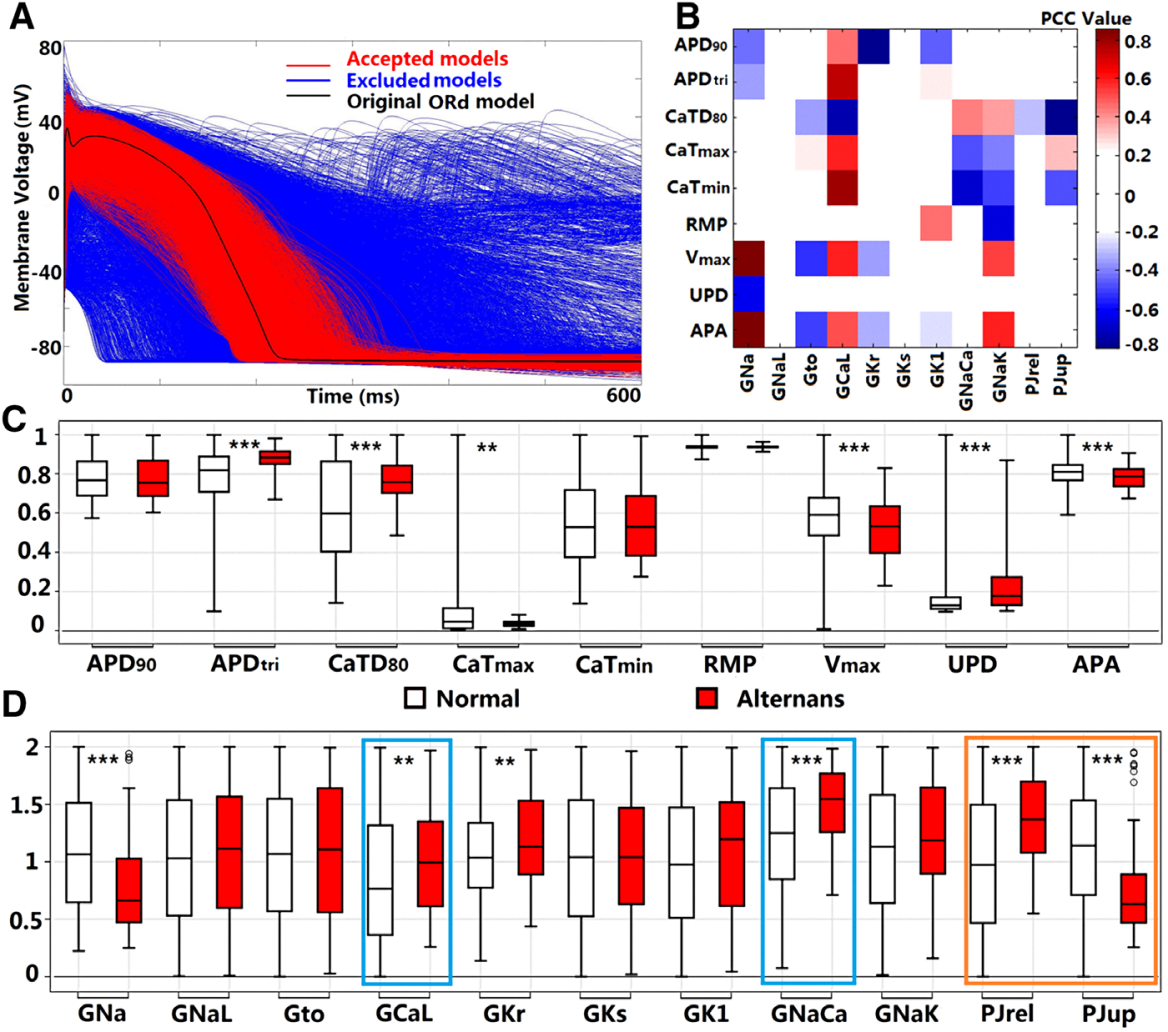
**Population of human ventricular models calibrated with in vivo recordings**. **A**, Action potentials of accepted and rejected models for a cycle length (CL) of 600 ms. **B**, Partial correlation coefficients (PCC) between action potential biomarkers and current conductance parameters. **C**, Action potential biomarkers for normal and alternans models for a CL of 350 ms. Biomarker values have been normalized against maximum values in all accepted models. **D**, Distribution of ionic conductances scaling factors for normal and alternans models with respect to their original value in the ±100% range (0–2). Symbols indicate statistical significance levels (*P<0.05, **P<0.01, ***P<0.001). APA indicates action potential amplitude; APD, action potential duration; CaTD, calcium transient duration; CaT_max_, systolic Ca^2+^ level; CaT_min_, diastolic Ca^2+^ level; G_CaL_, Ca^2+^ channel permeability; G_K1_, K1 channel conductance; G_Kr_, Kr channel conductance; G_Ks_, Ks channel conductance; G_Na_, fast Na^+^ channel conductance; G_NaCa_, Na^+^/Ca^2+^ exchanger conductance; G_NaK_, Na^+^/K^+^ pump activity; G_NaL_, late Na^+^ channel conductance; G_to_, transient outward potassium channel conductance; ORd, O’Hara–Rudy dynamic model; P_Jrel_, Ca^2+^ release permeability via ryanodine receptor to cytoplasm; P_Jup_, Ca^2+^ uptake permeability via sarcoplasmic reticulum Ca^2+^ ATPase pump from the cytoplasm; RMP, resting membrane potential; UPD, upstroke duration; and V_max_, peak upstroke voltage.

Figure 2A shows the APs generated using the population of human ventricular models, with models excluded (in blue) and accepted (in red) after calibration with in vivo recordings. Of the initial 10 000 models, 2326 human ventricular models were accepted after calibration (including the original ORd), covering a broad range of potential ionic properties values (Figures III and IV in the Online Data Supplement).

Figure 2B shows the correlation analysis between individual ionic properties and specific AP biomarkers, and it demonstrates that AP properties were often the result of the interplay of several currents. The results are in agreement with established knowledge on the role of specific ionic currents on human electrophysiology: (1) large AP upstroke (V_max_ and upstroke duration) was related to large fast Na^+^ channel conductance, whereas AP amplitude was also affected by G_CaL_, Na^+^/K^+^ pump activity and smaller transient outward potassium channel conductance; (2) the resting membrane potential was mainly determined by K1 channel conductance and Na^+^/K^+^ pump activity; (3) higher cytosolic Ca^2+^ transient levels (CaT_max_, CaT_min_) were related to larger G_CaL_ and smaller Na^+^/Ca^2+^ exchanger conductance and Na^+^/K^+^ pump activity; (4) shorter CaT duration was related to large G_CaL_ and Ca^2+^ uptake permeability via SERCA from the cytoplasm; (5) AP triangulation was mainly determined by G_CaL_; (6) APD was positively correlated with G_CaL_ and negatively correlated with Kr channel conductance, fast Na^+^ channel conductance, and K1 channel conductance (Figure 2B).

The human models in the calibrated population were classified into the normal (2239 of 2326 models) and alternans (87 of 2326 models) groups. Figure 2C displays box plots of the 9 biomarkers for normal and alternans models. No significant differences in APDs were found between the normal and the alternans groups, indicating that these APD alternans were not related to prolonged APDs. In contrast, AP triangulation tended to be larger in the alternans group, which suggested that AP morphology may be an indicator of alternans propensity. Although CaT duration was longer, CaT_max_ was found to be significantly smaller in the alternans than in the normal models, which further suggested the importance of Ca^2+^ dynamics in the generation of alternans. In agreement with this, Figure 2D shows that alternans models exhibited larger Ca^2+^ release permeability via RyR to cytoplasm and smaller Ca^2+^ uptake permeability via SERCA from the cytoplasm, as well as larger G_CaL_, Kr channel conductance and Na^+^/Ca^2+^ exchanger conductance, and smaller fast Na^+^ channel conductance than the normal models.

### Two Types of Alternans Are Observed in Both In Vivo and In Silico Data

**Figure 3.**
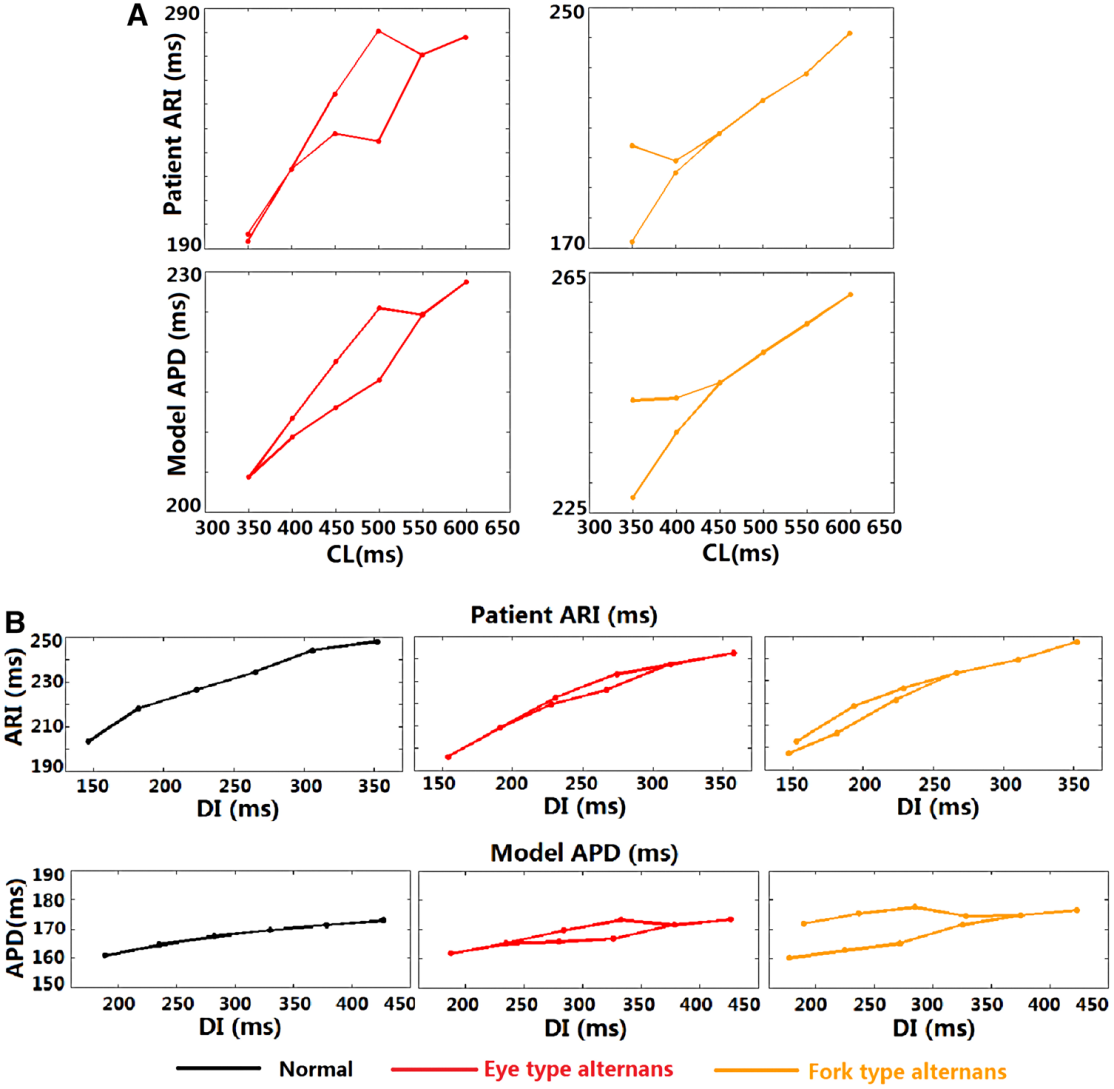
**Types of action potential duration (APD) alternans in vivo and in silico**. **A**, Representative restitution curves of APD (activation recovery interval [ARI]) vs cycle length (CL) exhibiting Eye-type and Fork-type alternans in vivo (**top**) and in silico (**bottom**) data. **B**, Representative restitution curves of APD (ARI) vs diastolic interval (DI) exhibiting normal condition and Eye-type and Fork-type alternans in vivo (**top**) and in silico (**bottom**) data.

The analysis of APD alternans in silico and in vivo revealed similar patterns, and in both cases, 2 types were identified as illustrated in Figure 3. Eye-type APD alternans were characterized by the disappearance of APD alternans at increasingly fast pacing rates (closed restitution bifurcation), whereas Fork-type APD alternans models displayed stable alternans at increasingly fast frequencies. In the in silico population of models, 14 human models displayed Eye-type restitution, and 47 models were Fork-type for the frequencies tested both in silico and in vivo. In silico, we were able to increase frequency to confirm that most Fork-type restitution curves (44 of 47) remained open when the pacing CLs were further decreased to 200 ms. In addition, 26 models displayed calcium alternans with APD alternans smaller than 5 ms in amplitude, named as CaT alternans models hereafter.

Both in silico and in vivo, APD alternans initiation started at longer CL in Eye-type alternans than in Fork-type alternans (median CL for APD alternans initiation: 550 and 500 ms in vivo, 475 and 350 ms in silico, respectively; with statistical differences *P*<0.001). Furthermore, both types of alternans occurred at similar diastolic interval and APD values than those exhibited by normal models (Figure 3B), which further supports the independence of APD alternans from APD or diastolic interval values. The fact that similar patterns of alternans are observed both in silico and in vivo recordings confers credibility to mechanistic investigations using the population of in silico human cardiomyocyte models.

### APD Alternans in the Human Ventricular Models Initiate After the Loss of SR Calcium Content Balance

**Figure 4.**
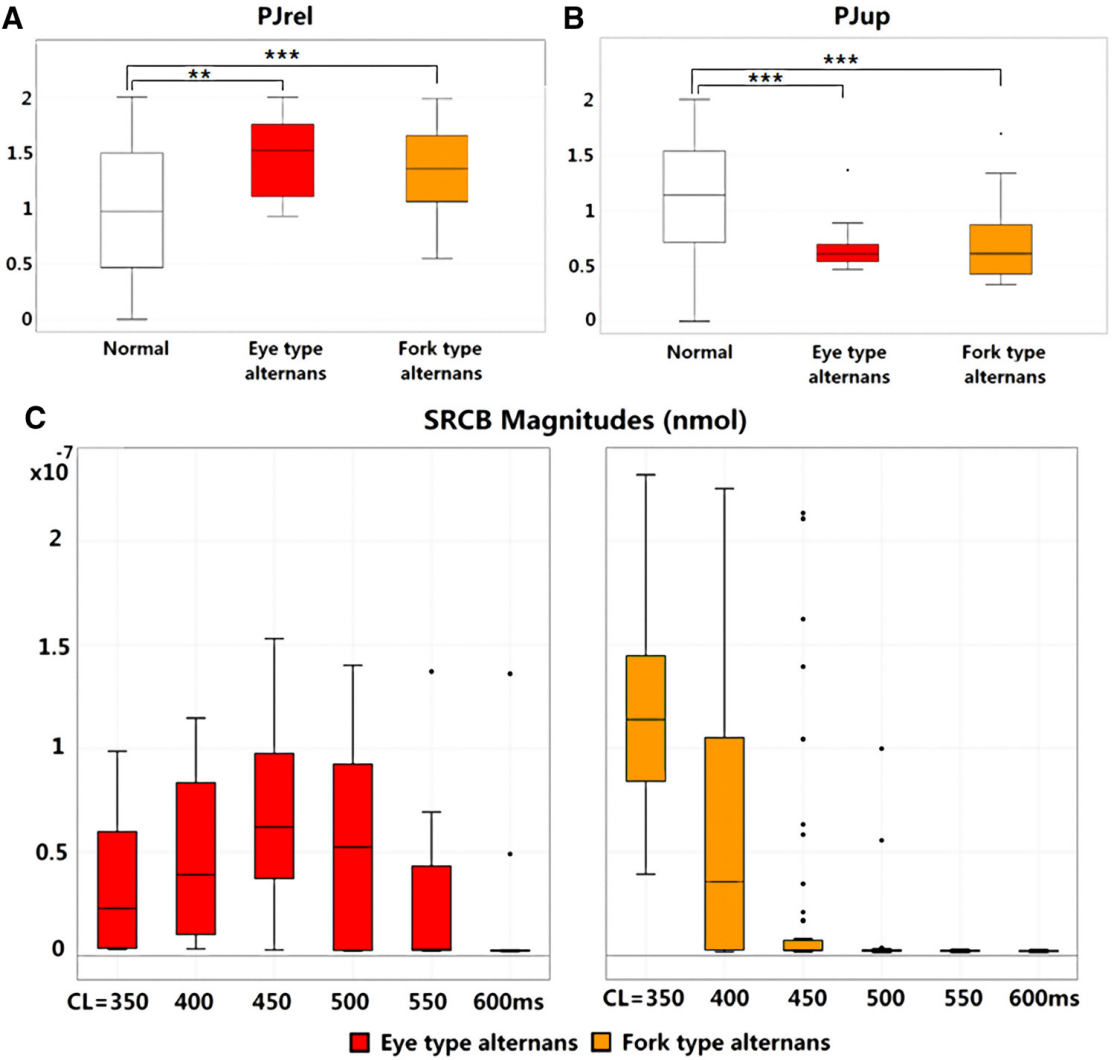
**SR Ca^2+^ cycling properties and fluctuations in alternans models**. **A** and **B**, Comparison of ryanodine receptor release (P_Jrel_, **A**) and sarcoplasmic reticulum Ca^2+^ ATPase pump uptake (P_Jup_, **B**) in normal, Eye-type, and Fork-type alternans models. The vertical axis shows parameters scaling. Symbols indicate statistical significance levels (*P<0.05, **P<0.01, ***P<0.001). **C**, Sarcoplasmic reticulum Ca^2+^ balance (SRCB) magnitudes for Eye-type and Fork-type alternans under all considered cycle lengths (CLs).

As shown in Figure 4A and 4B, both Eye-type and Fork-type alternans models displayed larger SR Ca^2+^ release (Ca^2+^ release permeability via RyR to cytoplasm) and smaller Ca^2+^ uptake (Ca^2+^ uptake permeability via SERCA from the cytoplasm) permeabilities than normal models. On the basis of these data, we hypothesize that APD alternans initiation in the in silico human cardiomyocytes is caused by fluctuations in junctional SR (JSR) calcium content because of the inability of SERCA (J_up_) to balance RyR Ca^2+^ release (J_rel_) at fast frequencies. If found in the human models, the mechanisms would be consistent with some previous measurements in rat and rabbit isolated cardiomyocytes.^11,12^

For all alternans models, the SR calcium balance (SRCB) was calculated as the integral of calcium ions uptaken by SERCA (J_up_) minus those released by RyR (J_rel_) over 1 beat at each CL (Online Table II). For both Eye-type and Fork-type models, SRCB magnitude displays beat-to-beat fluctuations during APD alternans, with 2 consecutive beats leading to similar SRCB magnitudes but of different sign (Online Figure V). Figure 4C shows the magnitude of SRCB for 1 short APD beat for each CL for Eye-type and Fork-type alternans models. For the CLs leading to APD alternans, SRCB magnitude increases above zero for both Eye- and Fork-type alternans models (Figure 4C), and its magnitude strongly correlates with the APD alternans magnitude (correlation coefficient ranging, 0.86–0.96 for all CLs).

The primary role of oscillations in calcium dynamics in generating APD alternans was confirmed by conducting simulated AP clamp experiments. We imposed the AP clamp of 2 identical long beats (L+L) and 2 identical short beats (S+S) to the Eye-type and Fork-type alternans models displaying the biggest alternans amplitudes (Online Figure VI). In the absence of APD alternans (imposed by the AP clamp), the Ca^2+^ alternans still persisted, which supported that the oscillations of SR Ca^2+^ content existed independently of APD alternans. Therefore, our results support that APD alternans in the human models initiate because of the fluctuations in SR calcium content, which was then transferred to the membrane potential as APD alternans.

### Strong I_NaCa_ and Fluctuations in SCB Result in APD Alternans in Both Eye-Type and Fork-Type Human Models, and Strong I_CaL_ Restores SCB Suppressing APD Alternans at Fast Pacing Rates for Eye-Type Models

**Figure 5.**
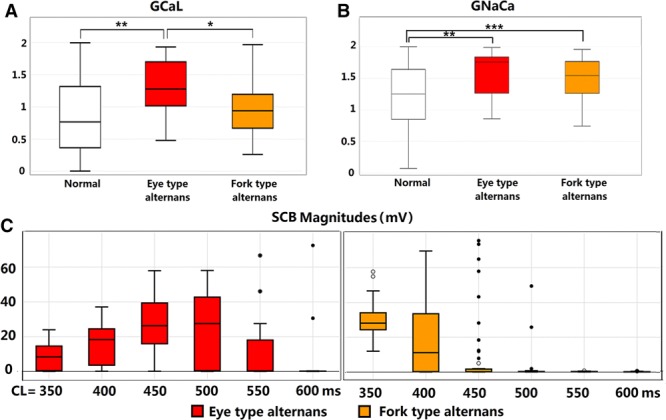
**Sarcolemmal Ca^2+^ balance (SCB) in alternans models**. **A** and **B**, Comparison of L-type Ca^2+^ channel conductance (G_CaL_, **A**) and Na^+^/Ca^2+^ exchanger conductance (G_NaCa_, **B**) in normal, Eye-type, and Fork-type alternans models. **C**, Sarcolemmal calcium balance (SCB) magnitudes for Eye-type and Fork-type alternans under all considered cycle lengths (CLs).

We then investigated the mechanisms underlying the translation from calcium alternans to APD alternans by further examining ionic differences between normal and alternans models. As shown in Figure 5A and 5B, the analysis of the in silico population reveals that the conductance of I_NaCa_ is significantly larger in both Eye-type and Fork-type models than in normal models, whereas the I_CaL_ conductance is larger in Eye-type models than its similar magnitude in Fork-type and normal models. A stronger I_NaCa_ in the human models would be expected to maximize the gain from calcium fluctuations to APD alternans, and this would be similar to findings in guinea pig myocytes.^31^ Therefore, we hypothesized that SRCB fluctuations destabilize the intracellular calcium balance, which then propagates to the membrane potential in the form of APD alternans through a strong I_NaCa_ in Eye-type and Fork-type models.

Figure 5C shows, for Eye-type and Fork-type alternans models, the SCB quantified as the integration of all the sarcolemmal calcium currents over 1 beat for each CL (Online Table II). As for SRCB, SCB magnitudes of the 2 alternating beats were practically equal but with different signs, which indicated that the overall calcium amount during the 2 beats was balanced (Online Figure VII). As for SRCB, a strong correlation was found between the magnitudes of SCB and APD alternans in Fork- and Eye-type alternans (correlation coefficient from 0.80 to 0.95).

The larger G_CaL_ in the Eye-type models resulted in stronger I_CaL_ and also larger CaT values than in Fork-type models, particularly for short CL <400 ms (Online Figure VIII). This leads to the enhancement of SERCA at fast frequencies, which allowed for restoring SRCB and suppressing SR content fluctuations at fast pacing rates for Eye-type models.

### Fine Balance in Sarcolemmal Currents, SR, and Intracellular Calcium Mechanisms Determines APD Alternans in Human Ventricular Myocytes

**Figure 6.**
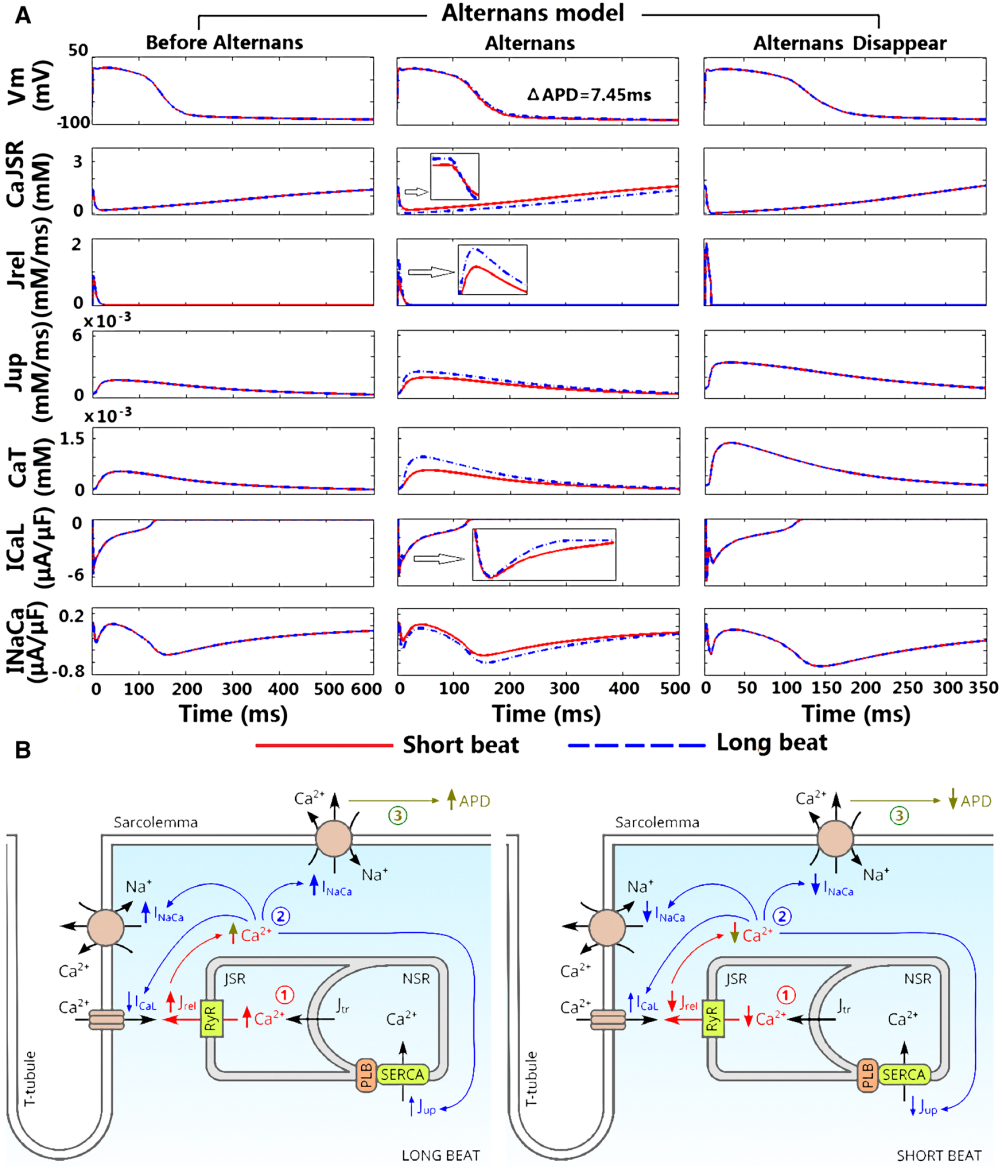
**Ionic mechanisms resulting in action potential duration (APD) alternans in human in silico ventricular cardiomyocytes**. **A**, From **top** to **bottom**, transmembrane potential (Vm), Ca^2+^ concentration in JSR (Ca_JSR_), Ca^2+^ release via ryanodine receptor (RyR; J_rel_), Ca^2+^ reuptake via sarcoplasmic reticulum Ca^2+^ ATPase pump (SERCA; J_up_), intracellular Ca^2+^ transient (CaT), L-type Ca^2+^ current (I_CaL_), and Na^+^/Ca^2+^ exchanger current (I_NaCa_) in a representative Eye-type model before the generation of alternans (cycle length [CL]=600 ms, **left**), during alternans (CL=500 ms, **middle**) and alternans disappearance (CL=350 ms, **right**). **B**, Schematic diagram illustrating the network of events explaining APD alternans for long and short beats: In (1), larger/smaller Ca_JSR_ levels at the start of the beat, results in larger/smaller J_rel_ and intracellular Ca^2+^ levels (2), leading to (3) increase/decrease in inward current through the I_NaCa_ forward mode, and a smaller decrease/increase in inward current through I_CaL_ calcium-induced inactivation. NSR indicates network SR; and PLB, phospholamban.

Figure 6 illustrates the network of events explaining alternans generation in the human ventricular myocytes. Figure 6A shows the time course of the transmembrane potential, JSR calcium level (Ca_JSR_), J_rel_, J_up_, intracellular CaT, and sarcolemmal calcium currents for 2 consecutive beats for a representative Eye-type model for a long CL with no alternans (CL=600 ms), for a fast CL resulting in alternans (CL=500 ms, middle column), and for a faster CL with no alternans (CL=350 ms, right column). Figure 6B provides a schematic representation of the ionic mechanisms involved, summarizing the ionic mechanisms for long versus short APD beats.

Beat-to-beat fluctuations in the magnitude of all properties are only observed in the middle panels of Figure 6A, as fast and slow pacing rates lead to alternans disappearance in the Eye-type model (left and right columns, respectively). During the long APD beat (blue dashed lines, first row), Ca_JSR_ (second row) reach a low level after SR release (third row), and then it progressively recovers because of SR reuptake (fourth row). However, the next beat starts before the Ca_JSR_ levels has reached its initial value, and this results in a lower Ca_JSR_ level at the start of the next beat (second row, compare red solid and blue dashed lines). The consequence for the next beat is a lower J_rel_ (third row, red solid lines), leading to a higher minimum Ca_JSR_ value (second row). The reuptake gradually recovers Ca_JSR_ content, which in this beat reaches a higher level at the end than at the start of the beat (red solid line, second row). The next beat would, therefore, start with higher Ca_JSR_ as in the blue dashed line, continuing the oscillations in Ca_JSR_ and SRCB as identified in Figure 4.

The beat-to-beat fluctuation in J_rel_ leads to intracellular CaT level oscillations (fifth row, middle column), which further results in the alternation of calcium related sarcolemmal currents such as I_CaL_ (sixth row, middle column) and I_NaCa_ (seventh row, middle column). For the beat with a higher initial Ca_JSR_ level and stronger J_rel_ (dashed blue lines), the amplitude of CaT is also higher. The fluctuation in intracellular Ca^2+^ content does not affect the I_CaL_ amplitude, in agreement with many studies showing that peak I_CaL_ is unchanged during alternans.^1,11,12^ However, it leads to a faster calcium-induced inactivation of I_CaL_ (sixth row), therefore, reducing the overall inward current. However, this is over-ridden by the calcium-induced potentiation of the forward-mode activity of I_NaCa_ (seventh row, middle column), which implies an increased inward current and results in longer APD (first row). I_NaCa_ is, therefore, the main electrogenic mechanism driving APD alternans in the human ventricular models.

The third column in Figure 6A illustrates the mechanisms underlying the disappearance of Ca_JSR_ fluctuations at faster CL for Eye-type models. As the CL is further decreased (third column), Ca^2+^ concentration increases because of the well-known Ca^2+^ accumulation at fast pacing rates, as reproduced by the models (fifth row). Increased Ca^2+^ levels enhance SR reuptake (fourth row) and speed up the recovery of Ca_JSR_ levels (second row), enabling for Ca_JSR_ levels to reach their initial values at the end of each beat. Therefore, fluctuations in Ca_JSR_ levels disappear at fast pacing rates as a result of rate-dependent calcium accumulation. Eye-type models display stronger I_CaL_ conductances than normal and Fork-type models, and this also results in larger intracellular calcium levels at fast pacing rates (Online Figure VIII). This is the reason why APD alternans are suppressed at fast pacing rates in Eye-type models.

Our simulations also explain the mechanisms underlying the occurrence of CaT alternans without significant APD alternans (<5 ms) in the 26 models. Calcium fluctuations were because of the same mechanisms as in the Eye-type and the Fork-type APD alternans models, but the magnitude of the oscillations in SRCB was smaller and did not result in significant APD alternans because of a modest Na^+^/Ca^2+^ exchanger conductance similar to the one in normal models.

### I_CaL_ Kinetics Variation Can Affect Alternans by Regulating SCB and SRCB

Given the role of I_CaL_ in modulating SCB and its importance in alternans generation, we investigated the effects of variations in I_CaL_ kinetics in modulating APD alternans and the SCB and SRCB. Simulations were conducted for varying I_CaL_ activation, inactivation, and recovery from Ca^2+^-dependent inactivation time constants in representative models, including the Eye-type and Fork-type models displaying the largest APD alternans. In this new set of simulations, we also considered the original ORd model and the 2 models in the normal population exhibiting the longest and shortest APD values, respectively. Variations in kinetics time constants of ±50% were considered to investigate theoretical mechanisms rather than representing specific pathological situations.

**Figure 7.**
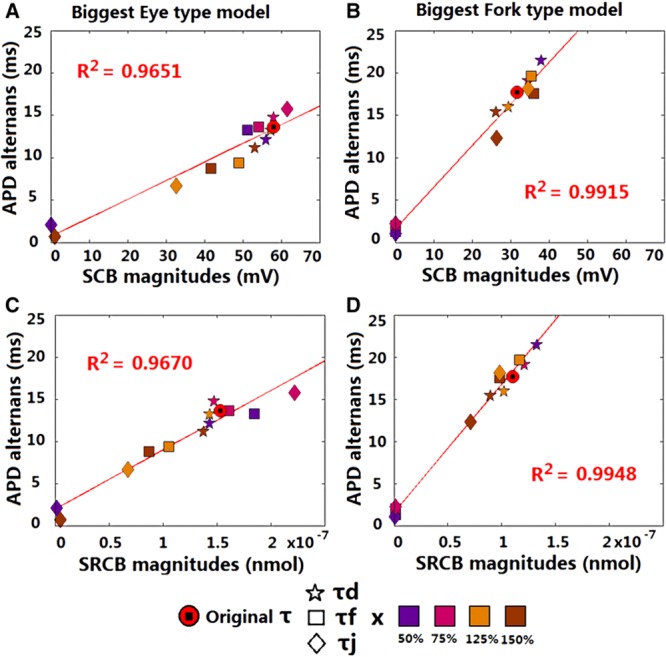
**Effects of varying L-type calcium current (I**_**CaL**_**) kinetics on the Eye-type and Fork-type models with biggest alternans magnitudes.** Correlation between action potential duration (APD) alternans magnitudes and sarcolemmal calcium balance (SCB) magnitudes in the Eye-type model with biggest alternans (**A**) and the Fork-type model with biggest alternans (**B**). Correlation between sarcoplasmic reticulum calcium balance (SRCB) magnitudes and APD alternans magnitudes in the biggest Eye -type model (**C**) and the biggest Fork-type model (**D**). Stars, squares, and diamonds represent the variation of I_CaL_ activation (τd), inactivation (τf), and recovery from Ca^2+^-dependent inactivation (τj) time constants, respectively. Colors represent changes in time constants magnitude. The red circle with a square inside represents the original kinetics.

Alternations of I_CaL_ kinetics did not produce alternans in any of the normal models considered. However, as shown in Figure 7, in both the Eye-type and Fork-type alternans models, variations particularly in I_CaL_ inactivation kinetics modulate the propensity of alternans generation. In all cases, APD alternans magnitude was still strongly correlated with SCB and SRCB (*R*^2^>0.96), further supporting the mechanisms unraveled in the previous sections. For the Eye-type model, slower I_CaL_ inactivation kinetics (increase in ICaL inactivation time constant and ICaL recovery from Ca^2+^-dependent inactivation time constant) decreased APD alternans because it increased an already strong I_CaL_, leading to further Ca^2+^ accumulation and increased SERCA activity, therefore, stabilizing SRCB and SCB. For the Fork-type model, however, the biggest effect was seen for fast inactivation (decrease in I_CaL_ inactivation time constant and I_CaL_ recovery from Ca^2+^-dependent inactivation time constant), which decreased APD alternans by decreasing I_CaL_ and consequently J_rel_, making it easier for SERCA to stabilize SRCB. The I_CaL_ conductance is, therefore, key to determining the effect of its inactivation kinetics in alternans generation, as it modulates the balance between the effect of I_CaL_ on both J_rel_ and the intracellular Ca^2+^ content, both of which are frequency dependent.

### I_NaCa_ Modulation Prevents APD Alternans in Human Ventricular Myocytes

**Figure 8.**
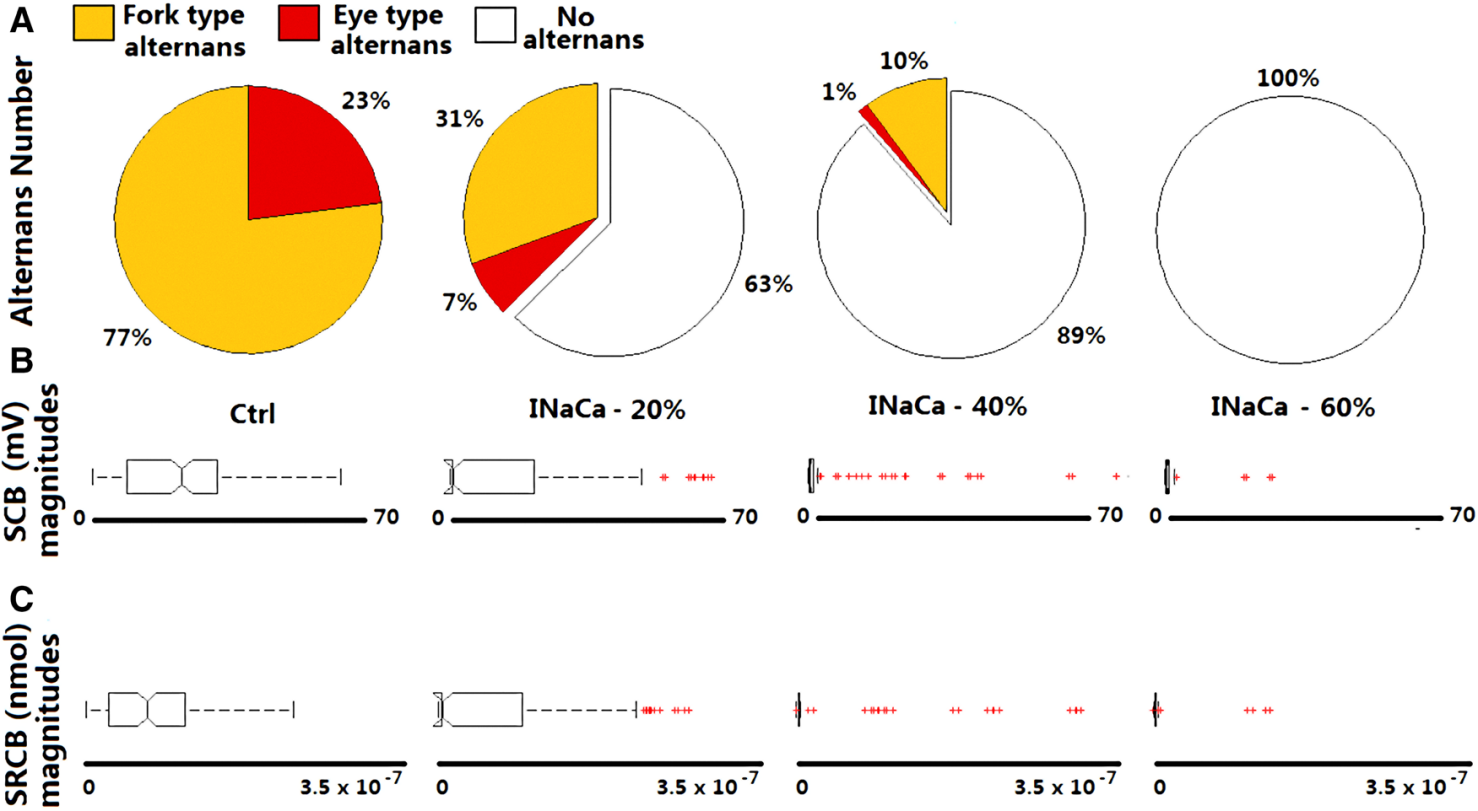
**Suppression of action potential duration (APD) alternans by Na^+^/Ca^2+^ exchanger current (I_NaCa_) inhibition**. **A**, Percentage of different types of alternans models under 20%, 40%, and 60% I_NaCa_ block. **B** and **C**, Effects of I_NaCa_ suppression on regulating the sarcolemmal calcium balance (SCB) and sarcoplasmic reticulum calcium balance (SRCB), respectively.

On the basis of our results, one of the fundamental events in the propagation of intracellular Ca^2+^ alternans to APD alternans is the extrusion of the over-released JSR calcium through I_NaCa_, which is stronger in alternans than in normal models. In addition, I_NaCa_ is also a crucial regulator of SCB, which is a fundamental indicator of alternans even after introducing I_CaL_ kinetics variation. Therefore, we explored the effects of suppressing the upregulated I_NaCa_ in all types of alternans models. Figure 8 shows the resulting percentage of alternans types and the change of SCB and SRCB after different I_NaCa_ interventions. Reducing the enhanced I_NaCa_ in alternans models by only 20% successfully converted 63% of the APD alternans models into normal models, whereas 60% I_NaCa_ reduction completely suppressed APD alternans (Figure 8A). I_NaCa_ modulation eliminated APD alternans by reducing the fluctuation in both SCB and SRCB (Figure 8B and 8C). In fact, I_NaCa_ inhibition only moderately shortens APD and increases the magnitude of the intracellular Ca^2+^ transient (Online Figure IXA and IXB). In addition, the maximum Ca^2+^ level in JSR also increased (Online Figure IXC).

## Discussion

In this in vivo and in silico human study, we unraveled the mechanisms and network of events leading to the occurrence of 2 types of APD alternans identified in novel human ventricular electrophysiological data. Variability in ionic conductances and permeabilities is shown to determine how human membrane kinetics translates calcium fluctuations into APD alternans. This study presents 2 main methodological novelties including the focus on human both in vivo and in silico, and the investigation of the mechanisms of frequency dependence of APD alternans without significant APD prolongation and long diastolic intervals. Our main new findings are as follows:

1. Both in vivo and in silico human ventricular cardiomyocytes reveal the existence of 2 types of APD alternans with Eye-type (closed bifurcation) and Fork-type restitution (open bifurcation) curves. Both types of APD alternans are observed for long diastolic intervals (>270 ms) and with normal (rather than prolonged) APD. Similarities between human in vivo and in silico alternans support the critical role of cellular processes of individual myocytes in RA generation and lend credibility to the computational investigations on the underlying mechanisms.
2. In the absence of APD prolongation, APD alternans in the in silico human cardiomyocyte population are consistently associated with fluctuations in SR Ca^2+^ content even taking into account ionic variability. The relative balance in flux densities between weak SERCA reuptake, strong RyR release, and strong I_NaCa_ extrusion determines the occurrence of alternans. Therefore, variability in ionic conductances and fluctuations do not explain alternative potential sources of Ca^2+^ alternans (such as RyR refractoriness), which still remain to be shown in human ventricular myocytes.
3. At increasingly fast frequencies, APD alternans disappear in Eye-type cardiomyocytes because of a strong I_CaL_, which leads to a frequency-induced increase in intracellular calcium levels that promotes SERCA and restores SRCB.
4. I_CaL_ conductance determines the effect of alterations in I_CaL_ inactivation kinetics in APD alternans, as it determines the balance between I_CaL_ effects directly on SCB and indirectly on SRCB through intracellular Ca^2+^ content and J_rel_.
5. Targeting I_NaCa_ sarcolemmal Ca^2+^ extrusion, as an indirect strategy to regulate intracellular Ca^2+^ cycling, successfully restores SR content balance and suppresses alternans generation in the human ventricular myocytes in agreement with previous rat and guinea pig studies (Online Table III), which supports the potential of I_NaCa_ as a promising antiarrhythmic target in human.

### Fluctuations in SR Ca^2+^ Content as a Primary Cause of APD Alternans in Human Ventricular Cardiomyocytes In Silico

Recent studies have reported that although there is a bidirectional coupling between membrane voltage and CaT, APD alternans tend to be the secondary consequence of Ca^2+^ cycling disturbances.^14^ The relationship between SR Ca^2+^ load and Ca^2+^ release on calcium alternans was proposed by Eisner et al.^32^ A steep Ca^2+^ release–SR Ca^2+^ load relationship was used to explain the generation of calcium alternans.^32^ Our in silico analysis also revealed weaker Ca^2+^ reuptake and stronger Ca^2+^ release in all types of alternans models (Figure 4), even considering variability in ionic conductances and permeabilities in the simulations. The role of SR Ca^2+^ reuptake in our results in human is consistent with the experimental observation that overexpression of SERCA2a suppresses alternans,^15,33,34^ whereas the suppression of SR Ca^2+^ release has also been shown to inhibit APD alternans in rabbit myocytes.^14^

In our human ventricular alternans models, fluctuations in SR Ca^2+^ content lead to Ca^2+^ and APD alternans, and this is in agreement with recordings in rat and rabbit isolated cell experiments.^11,13^ Recordings in rabbit myocytes and intact hearts^12,13^ have shown that in some cardiomyocytes, Ca^2+^ alternans can occur in the absence of SR Ca^2+^ content fluctuations because of RyR refractoriness during fast pacing. This was not observed in our human population triggering the following thoughts. First, new experiments are required to evaluate the potential contribution of RyR refractoriness to alternans in human ventricular myocytes. Second, the human population considered variability in ionic conductances and permeabilities, as well as frequency dependence of calcium dynamics. Indeed, the ORd model used to construct the population is able to reproduce key properties of Ca^2+^ cycling rate dependence as measured in human experiments, including frequency modulation of SR Ca^2+^ release, uptake, and content, modulated by Ca^2+^/calmodulin-dependent protein kinase II. However, SR Ca^2+^ content fluctuations were consistently observed during APD alternans. This suggests that if RyR refractoriness is shown to be a mechanism of RA in human in future studies, the in silico framework would need to be updated to reflect the new mechanisms, as it cannot be explained by differences in ionic protein expression in the current framework. Future experimental and theoretical studies are required to evaluate the need for updates in the complex calcium cycling framework, such as the calcium release units, to address the contributions of the 3R theory from calcium alternans to APD alternans.^35,36^

### SCB Translates Ca^2+^ Fluctuations Into APD Alternans in Human Ventricular Cardiomyocytes

Our human ventricular population shows that I_NaCa_ is larger in alternans than in normal models. Furthermore, we found that the increase of CaT amplitude and long APD beats were in phase (Figure 6), as was shown by Wang et al^13^ in intact rabbit hearts. An increase in CaT levels can induce both calcium-induced I_CaL_ inactivation (decrease inward current and shortening of APD) and increase of forward-mode I_NaCa_ (increase in inward current and prolongation of APD). Therefore, higher CaT corresponding to longer APD in our human models suggests that the forward-mode I_NaCa_ plays a more dominant role in prolonging APD for high calcium levels in the human ventricular myocytes, as in the study by Escobar and Valdivia.^37^ Our simulations suggest that I_NaCa_ modulation may effectively inhibit alternans occurrence in human ventricular cardiomyocytes. This is in agreement with the efficacy of I_NaCa_ block against Ca^2+^ oscillations and APD alternans in rat and guinea pig studies (Online Table III). Regulation of Ca^2+^ extrusion through I_NaCa_ may substantially differ in animals and human, and our study is the first human-based investigation to support the relevance of I_NaCa_ block potential against RA in human. Our in silico predictions could be further tested in future experimental studies, given in addition the recent availability of novel specific inhibitors of the sodium–calcium exchanger.^38^

Furthermore, interventions that promote the forward mode of I_NaCa_ may promote APD alternans. This can also be caused through its sensitivity to Na^+^, as, for example, described in a theoretical study using canine models which shown that suppressing fast Na^+^ current can produce larger APD alternans.^6^ In our simulation results, fluctuations in fast Na^+^ current and Na^+^ concentration were tightly linked with the alternation of I_NaCa_ during APD alternans, supporting the additional sensitivity of APD alternans to sodium content.

Even though the peak I_CaL_ does not fluctuate during APD alternans in the human ventricular models (as in experiments),^1^ we show that both conductance and kinetics of I_CaL_ modulate APD alternans in human ventricular myocytes both through the direct electrogenic effect of I_CaL_ on membrane potential and through indirect effects on intracellular Ca^2+^ content (which determines SR release and uptake). Strong I_CaL_ as in Eye-type models results in larger Ca^2+^ accumulation at fast pacing rates, which promotes SERCA and leads to restabilization of SR content and disappearance of APD alternans. Alterations in I_CaL_ inactivation kinetics can also modulate APD alternans, and their effect is different depending on the overall conductance of I_CaL_, as shown in Figure 7.

### Limitations

Rather than a single in silico model, the present study is built on a population-based in silico and in vivo approach, allowing the investigation of different alternans types in human ventricular cells and their common underlying mechanisms. Still, there are several limitations in this work: (1) in vivo information from aortic valve replacement or coronary artery bypass graft patients were used in this study, and we did not attempt to specifically model the pathologies of each patient. Instead, we varied the ionic properties of the ORd model in a wide range to investigate the contribution of variability in ionic conductances and permeabilities to explaining different APD alternans regimes. The in silico human models did predict similar alternans patterns at similar CLs to the in vivo data, supporting the validity of the methodology used. Although the authors believe that ORd is currently the best model for the purposes of this study, the findings might be model specific. (2) Only epicardial models and recordings were considered in the study because of the difficulties in acquiring simultaneous epicardial and endocardial recordings in vivo in human. (3) Although activation recovery interval is widely accepted as a surrogate for APD, as all indirect measurements, it may be affected by a bias. (4) In this study, we only considered the variability in ionic conductances and permeabilities. However, variability may also exist in current kinetics as a result of differences in protein structure and conformation (especially in the presence of genetic mutations). It is possible that alternans can also emerge from the kinetics of some currents, and, for example, RyR refractoriness as was shown in some rabbit ventricular cardiomycoytes.^12,13,36^ Further experiments would need to confirm the contribution of RyR mechanisms in human. (5) RA in whole-ventricles are caused and modulated by a variety of factors, including gap junctional coupling, tissue heterogeneity, and conduction velocity restitution (through, eg, fast Na^+^ current recovery from inactivation). Additional studies could focus on determining the interaction of the calcium-driven mechanisms of Eye-type and Fork-type alternans unraveled in our study with those additional factors in tissue.

## Acknowledgments

We thank Yoram Rudy for useful discussions and to acknowledge the use of the Advanced Research Computing in University of Oxford in carrying out this work.

## Sources of Funding

X. Zhou was supported by the China Scholarship Council. The UCL team was supported by UK Medical Research Council (G0901819), Marie Curie IEF-2013, and UCL Hospitals Biomedical Research Centre. B. Rodriguez and A. Bueno-Orovio were supported by BR’s Welcome Trust Senior Research Fellowship in Basic Biomedical Science (100246/Z/12/Z), an Engineering and Physical Sciences Research Council Impact Acceleration Award (EP/K503769/1) and the British Heart Foundation Centre of Research Excellence (RE/08/004/23915 and RE/13/1/30181).

## Disclosures

None.

## Supplementary Material

## Novelty and Significance

### What Is Known?

- Repolarization alternans are stable beat-to-beat fluctuations between subsequent action potentials (APs) and are regarded as an important risk factor for arrhythmogenesis.
- Animal experiments have revealed potential mechanisms of alternans associated with AP prolongation, fluctuations in sarcoplasmic reticulum (SR) calcium content and refractoriness in ryanodine receptors; however, characterization and investigation of alternans in human are lacking.
- Understanding sources and modulators of variability in human electrophysiology and alternans occurrence is a key challenge that requires alternative approaches to controlled laboratory techniques, which aim to suppress variability experimentally and statistically.
