# Supplementary material for: In Vivo and In Silico Investigation Into Mechanisms of Frequency Dependence of Repolarization Alternans in Human Ventricular Cardiomyocytes
Source: Circ Res. 2016 Jan 21;118(2):266–78. doi: 10.1161/CIRCRESAHA.115.307836 (PMC4719495; doi:10.1161/CIRCRESAHA.115.307836)
Supplement: Supplementary file 1 [file res-118-266-s001.pdf]

Supplemental Material

1. CHOICE OF MODEL OF HUMAN VENTRICULAR ELECTROPHYSIOLOGY

In silico investigations into the network of mechanisms underlying the generation of the two types of repolarization alternans in human ventricular cardiomyocytes were based on the O'Hara *et al.* model[1] (ORd). Even though several human ventricular models have been published in the past, the ORd model is now the gold standard for human studies of pro-arrhythmia, the only one extensively constructed and validated based on recordings over 140 human hearts, and the only model dimmed suitable for regulatory used by the USA Food and Drug Administration (see CiPA initiative). Importantly, the ORd model is the only one to include detailed formulations for the Ca^{2+} subsystem and Ca^{2+} extrusion dynamics based on human electrophysiology data. This is supported by the comparison to other models of the human ventricular action potential published prior to the ORd model, and specifically the well-known Grandi-Bers (GB) and Ten Tusccher-Panfilov (TP) models.[2,3] Table I summarises the key differences in intracellular Ca^{2+} regulation between the three models.

Human ventricular calcium cycling and extrusion	ORd	GB	TP
ICaL Ca^{2+}-dependent inactivation	Yes	Partly	No
Ca^{2+} TRPN buffering	Yes	Yes	No
Ca^{2+} CaMK buffering (ICaL, RyR, SERCA, ICaK, ICaNa)	Yes	No	No
Sarcoplasmic reticulum compartmentation	Yes	No	No
Human Na^{+}, Ca^{2+} and voltage dependence of INaCa Ca^{2+} extrusion	Yes	No	No

Table I: Calcium regulation in latest models of human ventricular electrophysiology. Yes/Partly/No refers to the presence of a given attribute in each of the considered models based on human data. TRPN: troponin; CaMK: Ca^{2+}/calmodulin-dependent protein kinase II; ORd: O'Hara *et al.* model[1] (2011); GB: Grandi *et al.* model[2] (2010); TP: ten Tusscher-Panfilov model[3] (2006).

As shown in Table I:

- Ca^{2+}-dependent inactivation (CDI) of ICaL is included in both the GB and the ORd models. However, the ORd model is the only one to include the formulation based on new recordings in undiseased human ventricular myocytes, in the presence of Ca^{2+} or Ba^{2+} as charge carriers to separate CDI from voltage dependent inactivation (VDI). The TP model only incorporates a VDI of ICaL.
- Both the ORd and GB models account for troponin cytosolic Ca^{2+} buffers. However, only the ORd contemplates the Ca^{2+}/calmodulin-dependent protein kinase II (CaMK) regulation of the human ventricular Ca^{2+} cycling, in particular through CaMK modulation of the ICaL current, sarcoplasmic reticulum (SR) Ca^{2+}-release through RyRs, and SR Ca^{2+}-reuptake by SERCA. CaMK buffering was found to play an important role for Ca^{2+} cycling, through the modulation of the Ca^{2+} transient amplitude, diastolic Ca^{2+} levels, rate dependence of junctional SR Ca^{2+} content and its evacuation, and magnitudes of RyR and SERCA Ca^{2+} fluxes.
- Only the ORd model contemplates a functional subdivision of the SR into a junctional (JSR) and network (NSR) compartments. In particular, the existence of a JSR subspace is of critical importance in calcium dynamics, as it serves as the effective Ca^{2+} pool sensed for the release of RyRs.
- Only the Na^{+}/Ca^{2+} exchanger of the ORd model, key for intracellular Ca^{2+} extrusion, has been formulated using measurements from undiseased human ventricular myocytes. Specifically, it allows for replicating the charge and Ca^{2+} flux reversal potentials of the exchanger, Na^{+} leak in the absence of Ca^{2+} exchange, and the Na^{+}, Ca^{2+} and voltage dependent properties of the INaCa current as observed in the non-failing human ventricle.

2. BIOMARKER CALCULATION OF SIMULATED ACTION POTENTIALS

The stimulation protocol in the simulations mimicked the one applied *in vivo*. *In silico* models were stimulated for 1000 beats at each of the CLs in a step protocol from 600ms to 350ms to reach their steady states. For each of the human models, the following biomarkers were calculated at steady state for each of the 6 considered CLs: APD (at three repolarization levels: APD_{30}, APD_{80}, APD_{90}), APD_{tri} (triangulation), CaTD (calcium transient duration), UPD (upstroke duration), V_{max} (peak upstroke

voltage), RMP (resting membrane potential), APA (action potential amplitude), CaT_{max} (systolic Ca^{2+} level) and CaT_{min} (diastolic Ca^{2+} level). See Supplemental Table II for a detailed description of biomarkers calculation. Peak current and flux magnitudes were calculated as the maximum absolute value of their current densities during the AP. We also calculated two additional property referred to as sarcoplasmic reticulum calcium balance (SRCB) and sarcolemmal calcium balance (SCB), defined as the overall Ca^{2+} flow through the SR and cell membrane, respectively (Supplemental Table II). The occurrence of APD (or CaTD) alternans was defined as a difference greater than 5ms between APD_{80} (or $CaTD_{80}$) in the last two APs of the pacing train.

Biomarker	Calculation method
RMP (resting membrane potential)	Minimum membrane voltage
V_{max} (peak upstroke voltage)	Maximum membrane voltage
APA (action potential amplitude)	V_{max}-RMP
Upstroke potential	RMP +APA*90%
Transmembrane threshold (at 30, 80 or 90% repolarization level)	RMP+APA*(100-repolarization level)%
UT (upstroke time)	Time at which membrane voltage rises to Upstroke potential
DT (depolarization time)	Time at which membrane voltage rises to Transmembrane threshold
RT (repolarization time)	Time at which membrane voltage falls back to Transmembrane threshold
UPD (upstroke duration biomarker)	UT-DT
APD (at 30, 80 or 90% repolarization level)	RT-DT
DI (diastolic interval)	cycle length – APD_{90} of the previous beat
APD_{tri} (triangulation biomarker)	APD_{30}/APD_{80}
CaT_{min} (diastolic calcium level)	Minimum value of intracellular Ca^{2+} transient concentration
CaT_{max} (systolic calcium level)	Maximum intracellular Ca^{2+} transient concentration
CaTD (at repolarization level 30 or 80)	Similar to APD, but based on intracellular Ca^{2+} transient
Initial alternans cycle length (CL)	The longest CL for alternans occurrence
Sarcolemmal calcium balance (SCB)	$SCB = \int [-(ICaL + IpCa + Icab) + 2 \times INaCa]\, dt$
SCB magnitude	Absolute value of SCB
Sarcoplasmic reticulum calcium balance (SRCB)	$\mathrm{SRCB}=\int (\mathrm{Jup} * \mathrm{Vcell\_NSR} - \mathrm{Jrel} * \mathrm{Vcell\_JSR})\, \mathrm{dt}$
SRCB magnitude	Absolute value of SRCB

Table II: Calculation of action potential biomarkers.

3. CALIBRATION OF THE HUMAN *IN SILICO* MODELS POPULATION

With our methodology, we specifically propose the investigation of variability in *in vivo* human ventricular rate dependence using *in silico* investigations with the population of human ventricular models. The *in vivo* recordings were analysed and the histograms shown in Supplemental Figure I show the distribution of ARI values for each of the CLs from 600 to 350ms. A rigorous analysis of the data was conducted, as described in Supplemental Figure II, to obtain physiological ranges of ARI variability *in vivo* for each CL while avoiding including possible outliers. This was done by fitting the aggregated ARIs at each CLs to a skewed normal distribution, and the cumulative distribution function was used to obtain 95% physiological ARI ranges to exclude the effects of extreme values but to still consider the variability in the data, as one of the key goals of our study.

The initial 10000 models in the human population were then filtered to only retain the models yielding APD values within the 95% in vivo ARI ranges for each of the CLs (Filter 1). In addition, we also ensured that each of the APD restitution curves was monotonically decreasing until the eventual occurrence of alternans (Filter 2). This calibration for rate dependence based on the *in vivo* data was crucial as it allowed retaining critical information on *in vivo* human rate dependence in the *in silico* study, an aspect that is key for the study of repolarization alternans.

In addition to calibrating the in silico population with the human *in vivo* rate dependence data (Filters 1 and 2), we also considered the following filters based on well-known properties of undiseased human ventricular cardiomyocytes reported in the literature:

- Filter 3 (resting potential in undiseased human ventricular cardiomyocytes): RMP between -100 and -64mV (thresholds computed as mean ± 2SD of experimental data by Li *et al* [4]).
- Filter 4 (upstroke amplitudes in undiseased human ventricular cardiomyocytes): V_{max} greater than 0mV.
- Filter 5 (upstroke duration in undiseased human ventricular cardiomyocytes): UPD smaller than 10ms.
- Filter 6 (resting Ca^{2+} levels in undiseased human ventricular cardiomyocytes): CaT_{min} between 21 and 285nM (thresholds computed as mean ± 2SD of experimental data by Piacentino *et al* [5]).

It is also important to stress that our approach does not aim to find a 1:1 match between the *in silico* and *in vivo* data, but rather to provide a tool to explore variability in human electrophysiology. This means that for example, a same model could indeed be representative of the rate dependent behavior of several sites *in vivo*. With the calibration with ensure that the human models in the population are representative of physiology variability in the *in vivo* data and cover a wide range of possible underlying combinations of ionic properties as illustrated Supplemental Figure III and analysed in the main body of the Manuscript. Supplemental Figure IV shows the effect of each of the Filters in constraining the distribution of each of the ionic properties. The consideration of a wide range of variability adds two advantages to the study. Firstly, we evaluate the consistency of the mechanisms of different alternans types when variability in ionic currents is considered within the population. Secondly, the calibrated population supports the model independency of the findings with respect to the parameter values, which is often compromised in studies using a single action potential model. Indeed in our study, we use over 2000 models to investigate the consistency in the mechanisms of alternans in human, all of them displaying physiological human electrophysiology consistent with the *in vivo* recordings too.

4. CALCULATION OF *IN VIVO* ALTERNANS AND *IN VIVO* RESTITUTION CURVES

The ventricles were stimulated from the apex of the left ventricle. The pattern of activation was consistent for different cycle lengths, being the correlation between the activation sequences for different S1 very high. In unipolar electrograms recorded *in vivo*, restitution curves illustrating Eye or Fork-type alternans are constructed as follows:

Definition of alternans: APD alternans was identified as being present whenever the beat-to-beat variation of ARI, $\Delta ARI = ARI_i - ARI_{i-1}$, exhibited an alternating pattern (long, short, long, short, etc) for at least 7 consecutive beats. Alternans magnitude was then calculated as median ($|\Delta ARI_k|$) where k represents the heart beats exhibiting an alternating pattern.
The first 7 beats of each train of steady state S1 paced beats are discarded in order not to include alternans due to fast rate adaptation.

Classification of Eye/Fork types of alternans

1. Recordings are divided in recordings showing alternans at least at one cycle length (alternans susceptible site) and recording not showing alternans (alternans resistant site).

2. Recordings corresponding to alternans-susceptible sites are divided into Fork/Eye type:
   a. A site is said to show Fork-type alternans if alternans are present at CL=350ms.
   b. A site is said to show Eye-type alternans if alternans are not present at CL=350ms.

Eye/Fork alternans restitution curve plots
For each site (i.e. channel, electrode etc) ARIs are shown for each cycle length.

- If at a given cycle length there are no alternans, only one point is plotted: this point corresponds to the median ARI.
- If at a given cycle length alternans occur then two points are plotted. These 2 points correspond to $ARI_m \pm ALT/2$, where ARI_m is the median ARI calculated over the ARI that are actually alternating, and ALT is the alternans magnitude. The span between the 2 points is equal to the alternans magnitude.

Initial alternans CL for *in vivo* Eye/Fork alternans: the longest pacing cycle length when alternans occur at a site.

5. ACTION POTENTIAL CLAMP SIMULATION

For each single model, two protocols were generated from its steady state: two consecutive long beats (L+L) or two consecutive short beats (S+S). The simulation started from the end of the 1000th beat, and then either the L+L protocol or the S+S protocol were applied.

6. ICAL KINETICS VARIATION ANALYSIS

I_{CaL} activation time constant τd, inactivation time constant τf and recovery from Ca^{2+} dependent inactivation time constant τj were decreased (×50% or 75%) or increased (×125% or 150%), and models were still paced for 1000 beats to reach steady states.

7. PREVIOUS STUDIES ON Na^+/Ca^{2+} EXCHANGER BLOCK AND ALTERNANS

Authors	Species	Technique	I_{NaCa} blocker	Main conclusions
Schäfer *et al.*[6] (2001)	Rat myocytes	Wet lab	KB-R7943 (reverse-mode blocker)	Inhibition of the reverse mode I_{NaCa} reduced spontaneous Ca^{2+} oscillations upon reperfusion.
Satoh *et al.*[7] (2003)	Rat and guinea pig myocytes	Wet lab	KB-R7943 (reverse-mode blocker)	I_{NaCa} is species-dependent, and blocking reverse-mode I_{NaCa} abolished spontaneous Ca^{2+} oscillations upon ischemia/reperfusion.
Wan *et al*[8] (2012)	Guinea pig myocytes	Wet lab / Modelling	SEA-0400	Coupling from CaT alternans to APD alternans is determined by the relative balance between I_{NaCa} and I_{CaL}. Under control condition, I_{NaCa} is the major coupling link.

Table III: Previous studies on I_{NaCa} block related to Ca^{2+} and APD alternans.

8. SUPPLEMENTAL FIGURES

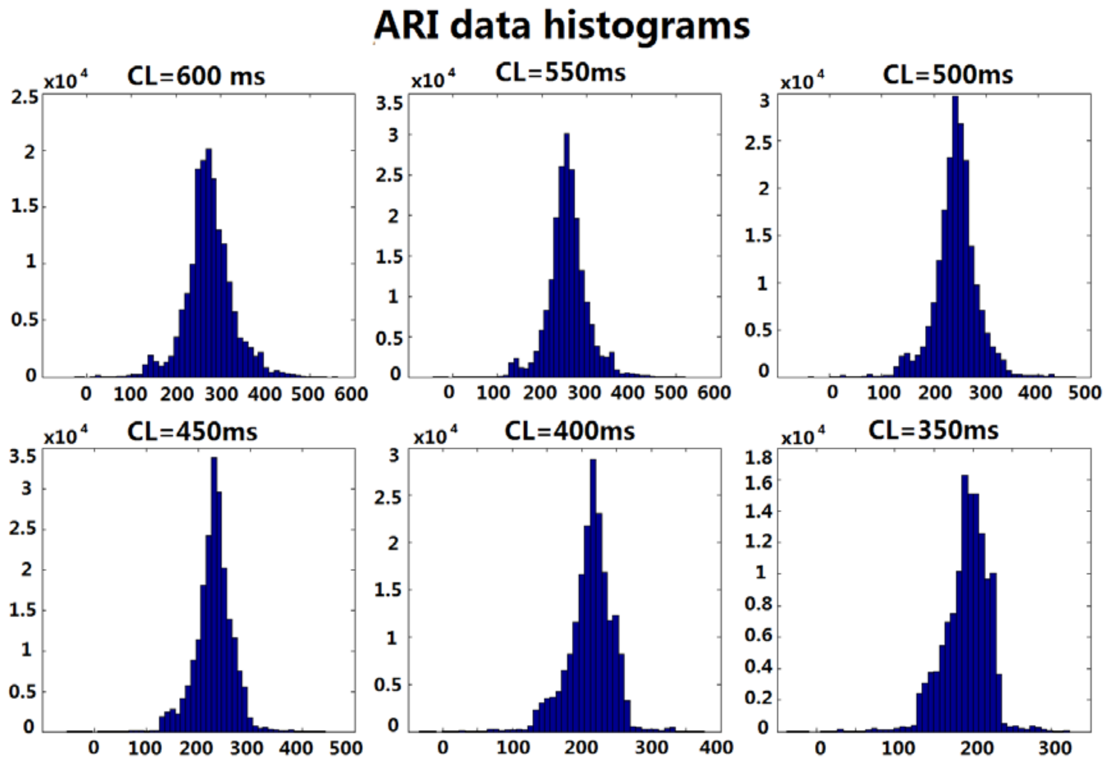

Supplemental Figure I: Distribution of *in vivo* ARI data from patients. Aggregated ARIs at the different CLs presented a skewed distribution

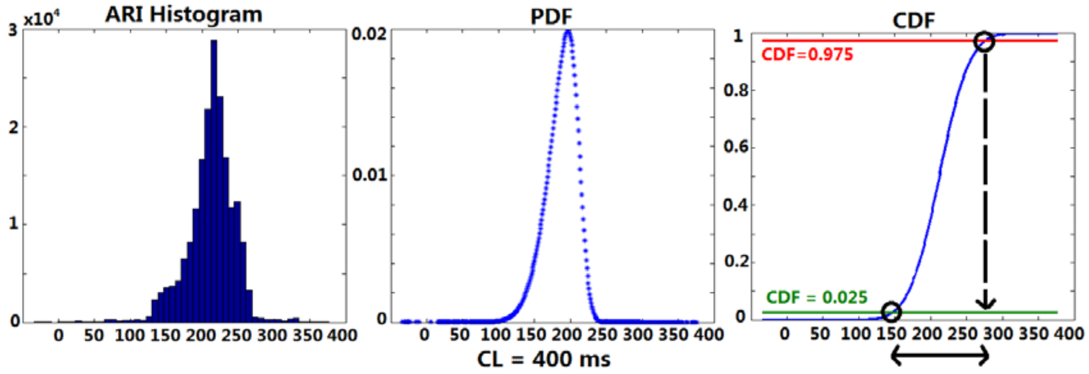

Supplemental Figure II: Probabilistic analysis of *in vivo* ARI data. Aggregated ARIs at the different CLs were fitted by a skewed normal distribution. The cumulative distribution function was used to obtain 95% physiological ARI ranges to exclude the effects of extreme values. Left: histogram of ARI data from all patients at a CL of 400ms. Middle: probability distribution function (PDF) of the original data based on a skewed normal distribution. Right: Selection of 95% data coverage thresholds (black circles) in a reconstructed cumulative distribution function (CDF).

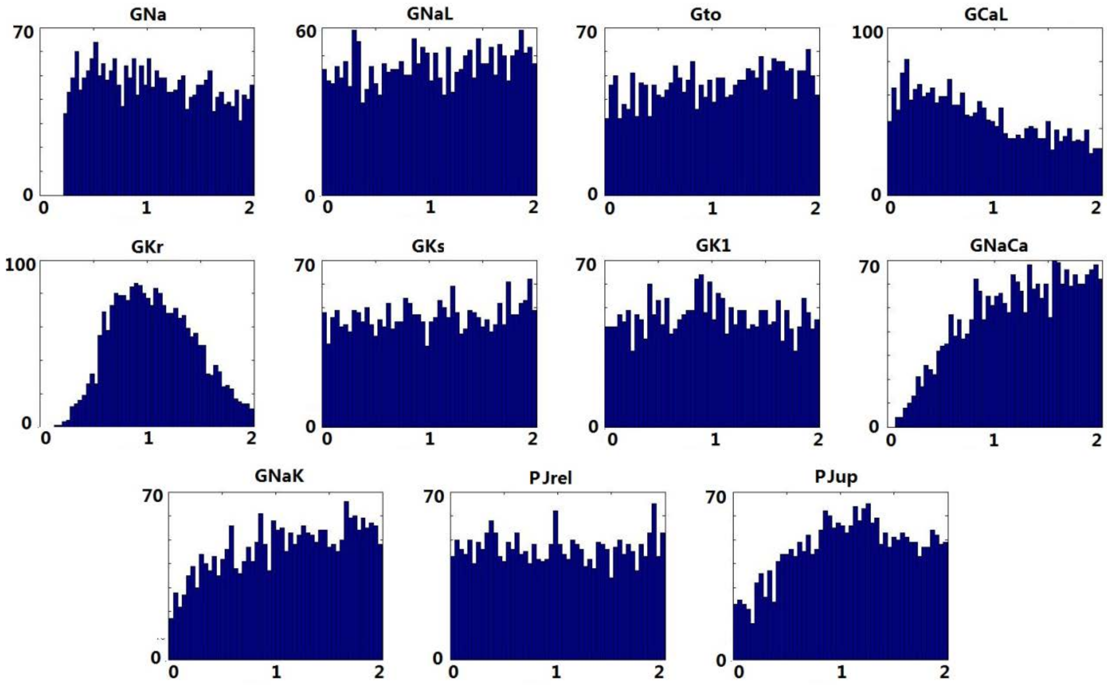

Supplemental Figure III: Histograms of accepted conductance parameters. The distribution of several ionic parameters tended to be asymmetric, such as those for G_{CaL}, G_{NaCa}, G_{NaK} and P_{Jup}, whereas the distribution of G_{Kr} was bell-shaped. Small values in G_{Na} were completely rejected after the calibration, which indicated the irreplaceable role of this Na^{+} current in the action potential upstroke.

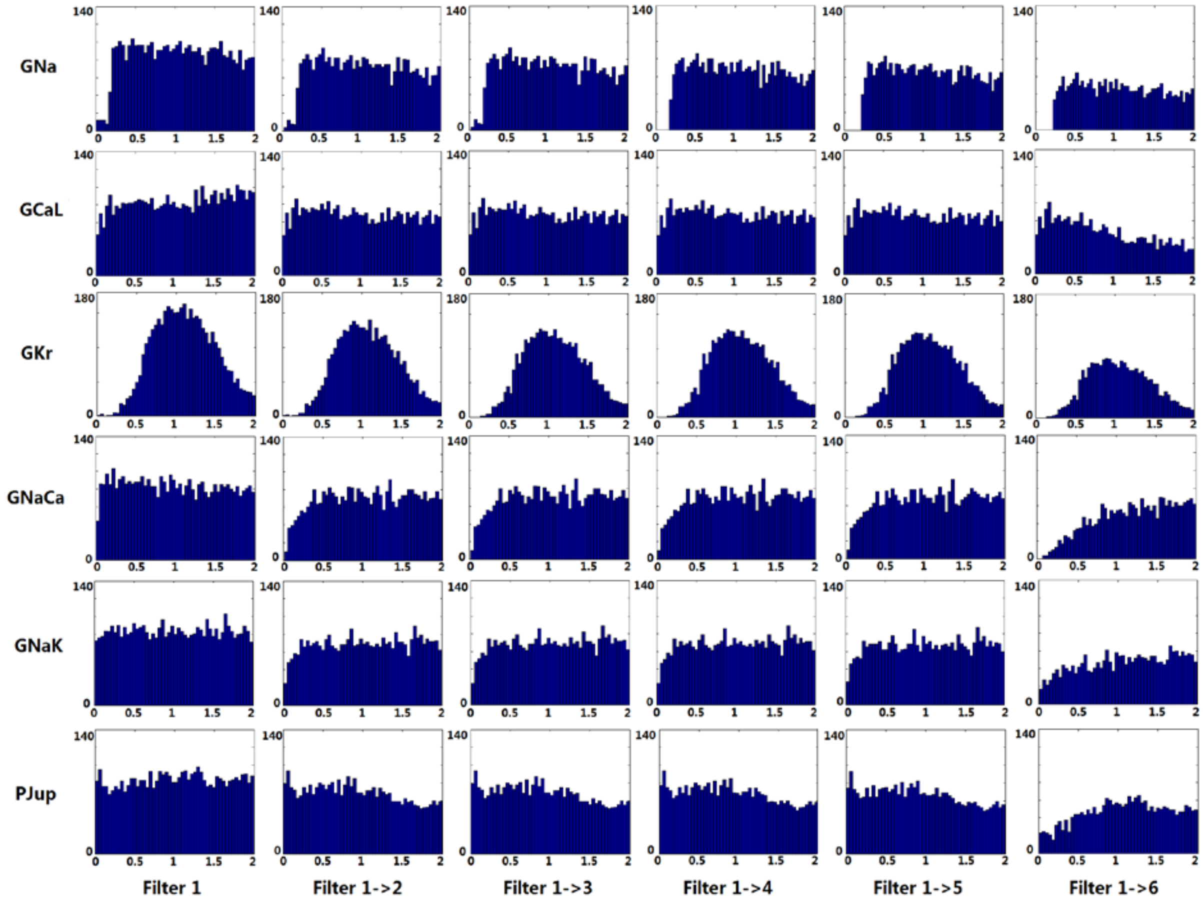

Supplemental Figure IV: Distribution of ionic properties in the population of human models following the different calibration filters. Each column shows from consideration of first Filter 1 to addition of each of the consecutive filters:

- Filter 1 (*in vivo* ARI value ranges): APD_{90} within the 95% physiological ARI ranges calculated from all patients under each CL.
- Filter 2 (*in vivo* ARI rate dependence): APD_{90} restitution within the 95% physiological envelope of ARI restitution as calculated from all patients, ensuring a monotonically decreasing restitution curve in all models as CL decreases until alternans occurrence.
- Filter 3 (resting potential in undiseased human ventricular cardiomyocytes): RMP between -100 and -64mV (thresholds computed as mean ± 2SD of experimental data by Li *et al* [4]).
- Filter 4 (upstroke amplitudes in undiseased human ventricular cardiomyocytes): V_{max} greater than 0mV.
- Filter 5 (upstroke duration in undiseased human ventricular cardiomyocytes): UPD smaller than 10ms.
- Filter 6 (resting Ca^{2+} levels in undiseased human ventricular cardiomyocytes): CaT_{min} between 21 and 285nM (thresholds computed as mean ± 2SD of experimental data by Piacentino *et al* [5]).

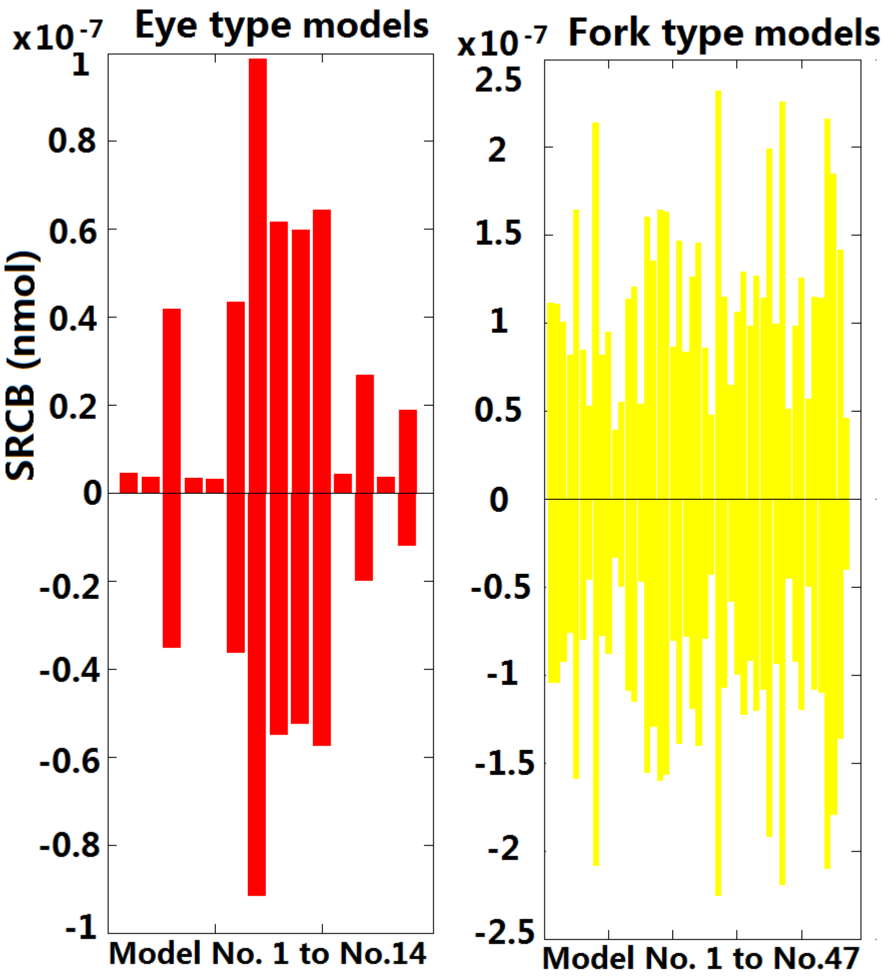

Supplemental Figure V: SRCB analysis between long/short alternating steady-state beats. Sarcoplasmic reticulum Ca^{2+} balance (SRCB) of the final two alternating beats in all APD alternans models (CL=350ms). The SRCB in long and short beats compensates for each other (same magnitudes with opposite signs).

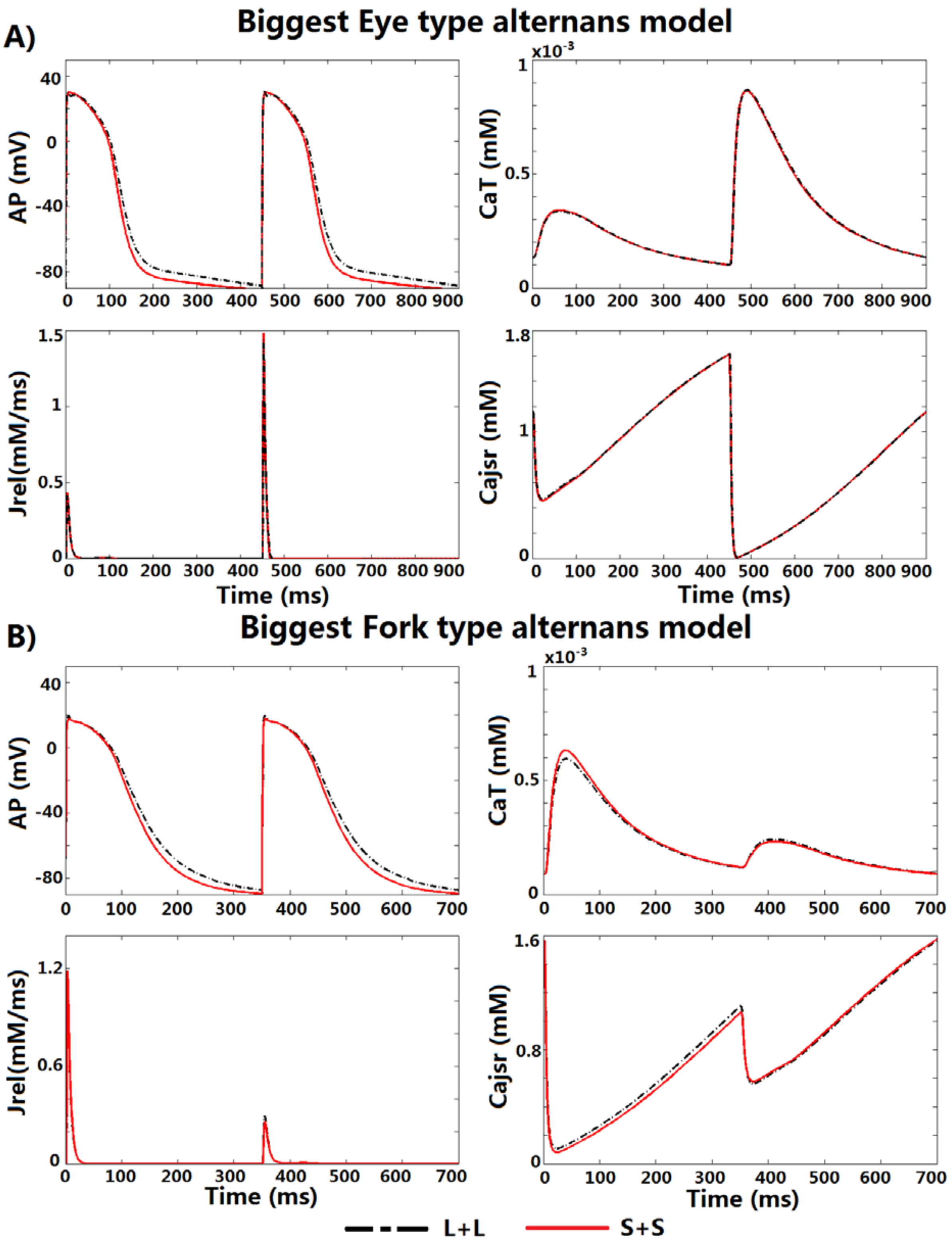

Supplemental Figure VI: Action potential clamp simulations. Two identical long beats (L+L) or two identical short beats (S+S) were applied to Eye-type (**A**) and Fork-type (**B**) alternans representative models with biggest alternans amplitude.

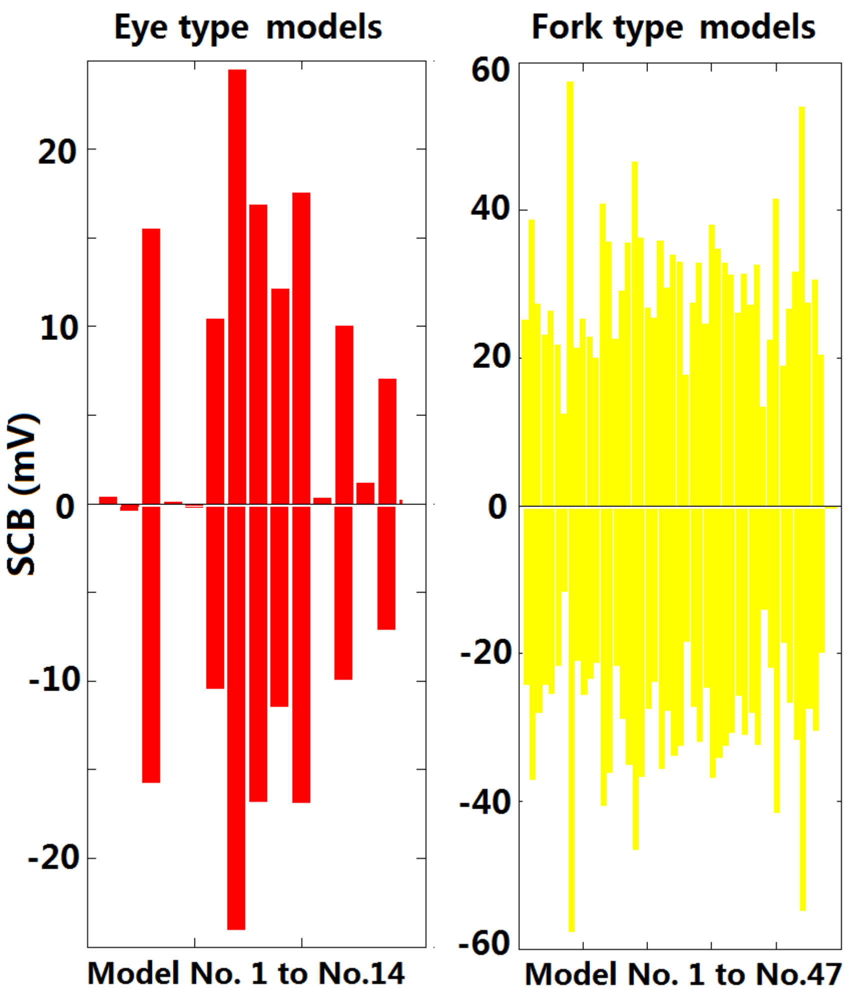

Supplemental Figure VII: SCB analysis between long/short alternating steady-state beats. Sarcolemmal Ca^{2+} balance (SCB) of the final two alternating beats in all APD alternans models (CL=350ms). The SCB in long and short beats compensates for each other (same magnitudes with opposite signs).

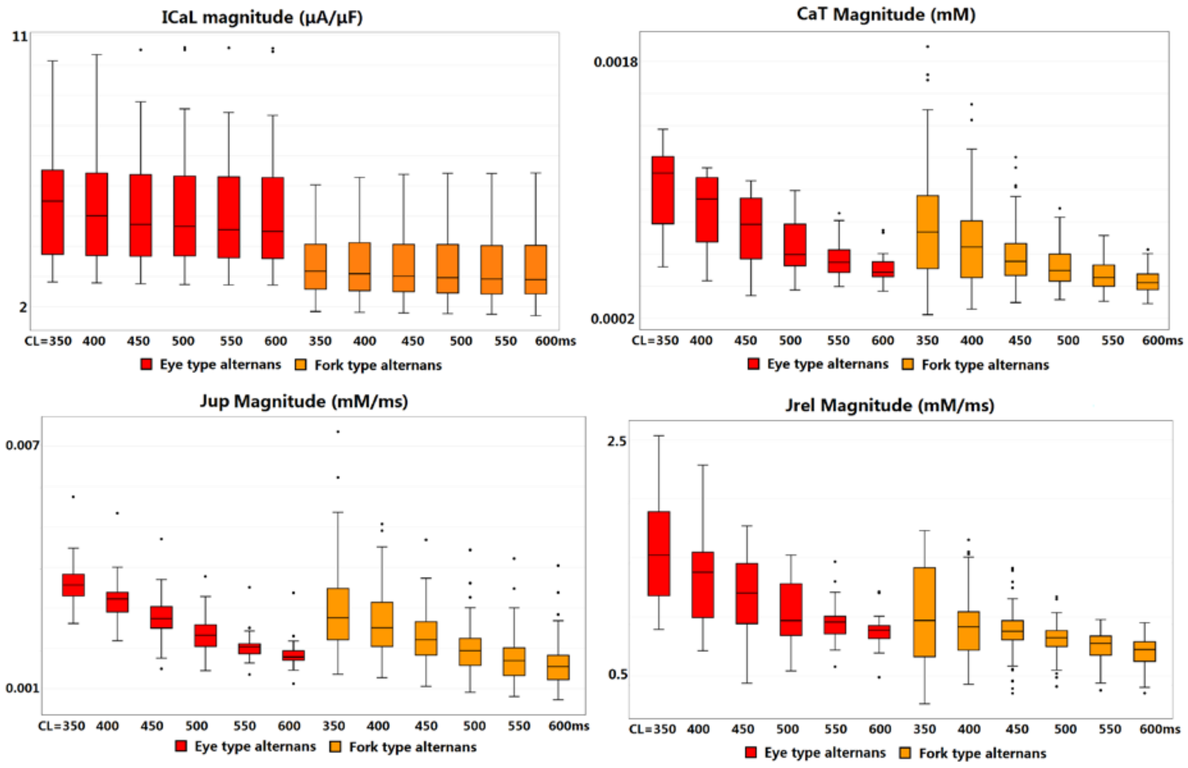

Supplemental Figure VIII: Rate dependency of ICaL magnitude, CaT magnitude, Jup magnitude and Jrel magnitude.

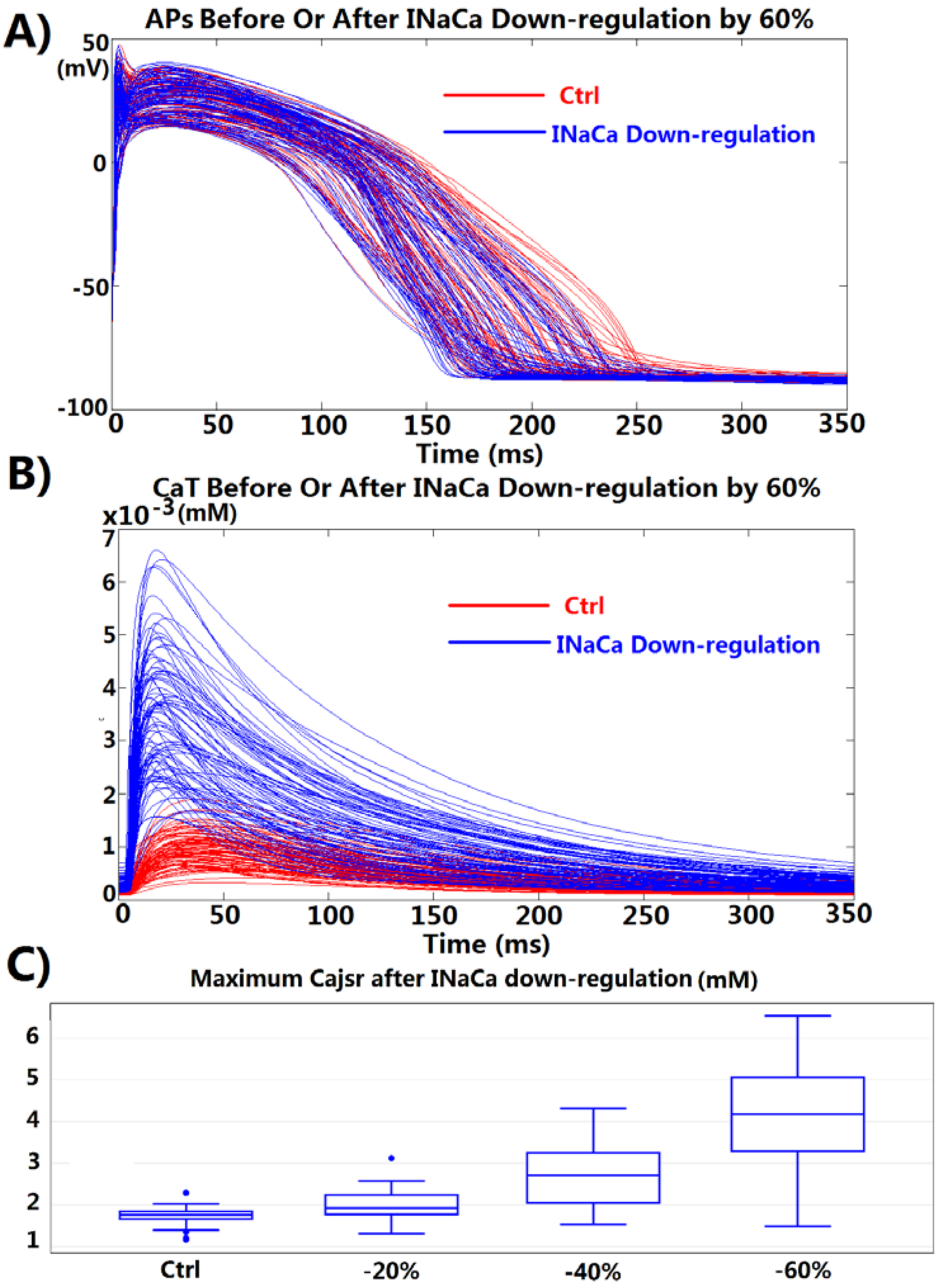

Supplemental Figure IX: Effects of I_{NaCa} down-regulation on AP, CaT and JSR Ca^{2+} in alternans models. A, B: Effects of I_{NaCa} inhibition by 60% on AP and CaT. **C:** Maximum JSR Ca^{2+} level in alternans models before and after I_{NaCa} inhibition at a CL=350ms.

SUPPLEMENTAL REFERENCES

1. O'Hara T, Virág L, Varró A, Rudy Y. Simulation of the Undiseased Human Cardiac Ventricular Action Potential: Model Formulation and Experimental Validation. *PLoS Comput Biol*. 2011;7:e1002061.

2. Grandi E, Pasqualini FS, Bers DM. A novel computational model of the human ventricular action potential and Ca transient. *J Mol Cell Cardiol*. 2010;48:112–121.

3. Ten Tusscher KHWJ, Panfilov AV. Cell model for efficient simulation of wave propagation in human ventricular tissue under normal and pathological conditions. *Phys Med Biol*. 2006;51:6141–6156.

4. Li G-R, Lau C-P, Leung T-K, Nattel S. Ionic current abnormalities associated with prolonged action potentials in cardiomyocytes from diseased human right ventricles. *Heart Rhythm*. 2004;1:460–468.

5. Piacentino V, Weber CR, Chen X, Weisser-Thomas J, Margulies KB, Bers DM, Houser SR. Cellular basis of abnormal calcium transients of failing human ventricular myocytes. *Circ Res*. 2003;92:651–658.

6. Schäfer C, Ladilov Y, Inserte J, Schäfer M, Haffner S, Garcia-Dorado D, Piper HM. Role of the reverse mode of the Na+/Ca2+ exchanger in reoxygenation-induced cardiomyocyte injury. *Cardiovasc Res*. 2001;51:241–250.

7. Satoh H, Mukai M, Urushida T, Katoh H, Terada H, Hayashi H. Importance of Ca2+ influx by Na+/Ca2+ exchange under normal and sodium-loaded conditions in mammalian ventricles. *Mol Cell Biochem*. 2003;242:11–17.

8. Wan X, Cutler M, Song Z, Karma A, Matsuda T, Baba A, Rosenbaum DS. New experimental evidence for mechanism of arrhythmogenic membrane potential alternans based on balance of electrogenic INCX / ICa currents. *Heart Rhythm Off J Heart Rhythm Soc*. 2012;9:1698–1705.
